# Supplementary material for: Evidence of a persistent altered neural state in people with fibromyalgia syndrome during functional MRI studies and its relationship with pain and anxiety
Source: PLoS One. 2025 Jan 24;20(1):e0316672. doi: 10.1371/journal.pone.0316672 (PMC11759356; doi:10.1371/journal.pone.0316672)
Supplement: S1 Appendix — (DOCX) [file pone.0316672.s001.docx]

**S1 Appendix: *Comparisons of BOLD responses detected with data from the brain and from the brainstem/spinal cord***

The BOLD time-course responses from these sub-regions that occur in both data sets (brain and brainstem/cord) were compared to ascertain whether or not these data sets provide similar information that can be combined. BOLD time-course responses in the locus coeruleus are shown in fibromyalgia (FM) and healthy control (HC) groups in Figure S1.1. Even though the BOLD responses were measured using different methods and different spatial and temporal resolutions, the time-courses are shown to have similar features. For example, data sets obtained with the two methods demonstrate an initial rise in signal, as well as signal variations when participants were informed of what to expect and while anticipating the stimulus. This was followed by relatively low signal variations during the stimulation period. Direct comparison of the time-series responses were obtained by first interpolating the values to the sampling rate of the brainstem/cord data (TR = 6.75 sec, 40 time points) and allowing time shifts up to ½ of a TR period to account for differences in slice timing. The time-series responses with the two methods were observed have correlation, R, values of 0.263 and 0.446 for HC and FM data, respectively (corresponding to p = 0.051 and p = 0.0018). The data therefore demonstrate similar BOLD response features and the two methods are expected to provide similar results with Structural and Physiological Modeling (SAPM).


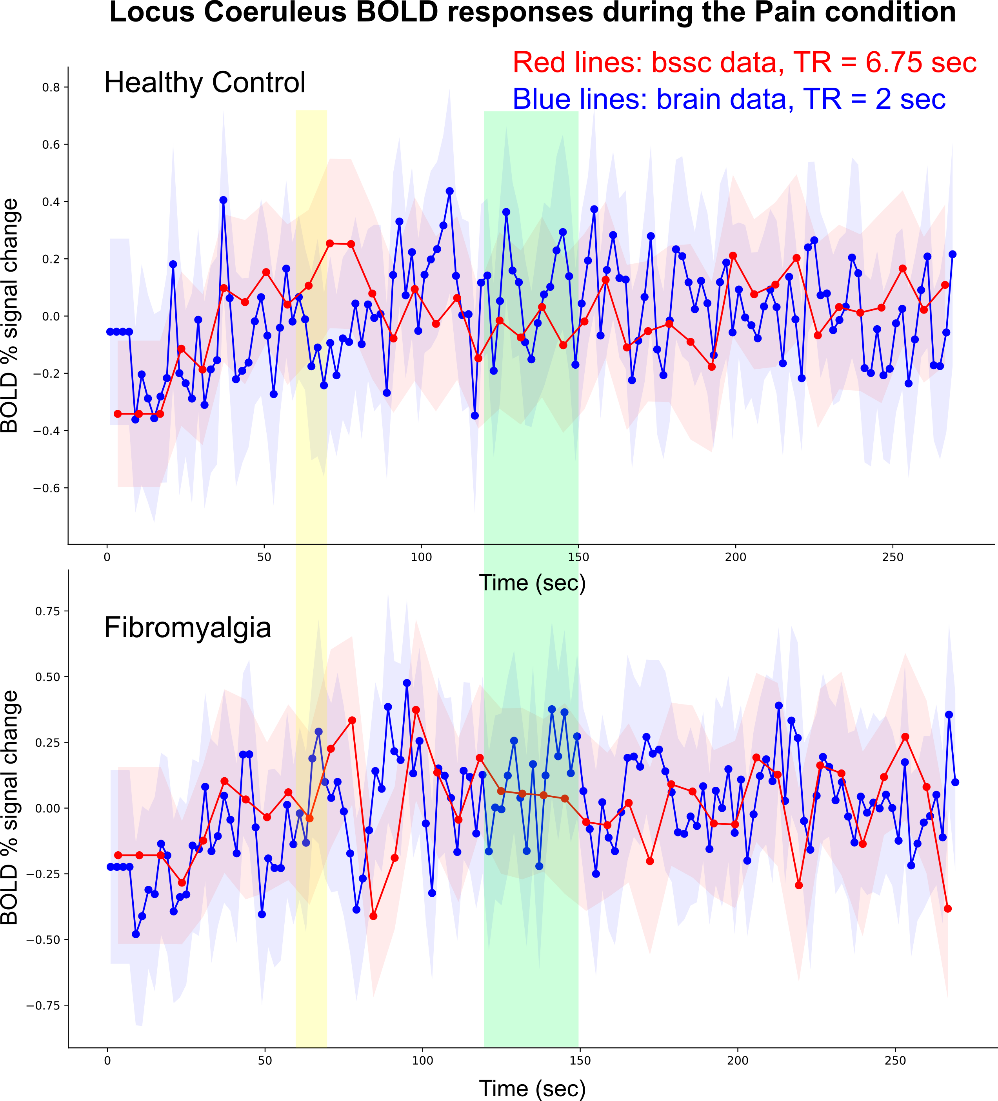


**Figure S1.1.** BOLD time-course responses in the locus coeruleus measured with brainstem/cord data using T_2_-weighted data (red lines), compared with BOLD responses measured using T_2_*-weighted data (blue lines). The top panel shows results obtained in healthy control participants (HC) and the bottom panel shows results from participants with fibromyalgia (FM). With data interpolated to the same TR interval as for the brainstem/cord data and allowing time shifts of less than TR/2 to account for diffferences in slice timing corrections, the correlation, R, between the time-series responses with the two methods are 0.263 and 0.446 for HC and FM data, respectively (corresponding to p = 0.051 and p = 0.0018). The period when participants were informed of the stimulus type is indicated with a yellow band, and the stimulation period is indicated with a green band.
