# Supplementary material for: Evidence of a persistent altered neural state in people with fibromyalgia syndrome during functional MRI studies and its relationship with pain and anxiety
Source: PLoS One. 2025 Jan 24;20(1):e0316672. doi: 10.1371/journal.pone.0316672 (PMC11759356; doi:10.1371/journal.pone.0316672)
Supplement: S2 Appendix — (DOCX) [file pone.0316672.s002.docx]

**S2 Appendix: *Details of all ANCOVA comparisons between study groups (FM vs HC), connectivity values and the magnitude of the initial rise in BOLD signal, in relation to questionnaire scores***

All ANCOVA comparisons were carried out with the group (FM, n = 20 vs HC, n = 17) as a categorical independent variable, and pain ratings or questionnaire scores as a continuous independent variable.

**Table S2.1:** Variations of connectivity (DB) values in relation to group (FM vs HC) and pain ratings (2-way ANCOVA)

| **Connection** | **Group 1**  **DB values** | **Group 2**  **DB values** | **Main effect of Group** | | **Main effect of**  **Pain Ratings** | | **Interaction Effect** | |
| --- | --- | --- | --- | --- | --- | --- | --- | --- |
|  | **(mean ± s.d.)** | **(mean ± s.d.)** | **F** | **p** | **F** | **p** | **F** | **p** |
| AC-IC | 0.029 ± 0.029 | -0.042 ± 0.022 | 6.8846 | 0.0135 | 7.0796 | 0.0124 | 5.1418 | 0.0307 |
| AC-PC | 0.402 ± 0.069 | 0.679 ± 0.066 | 6.4836 | 0.0163 | 0.1373 | 0.7135 | 0.2221 | 0.6409 |
| PC-Thal | 0.025 ± 0.031 | -0.089 ± 0.035 | 5.1099 | 0.0312 | 0.0150 | 0.9032 | 0.0627 | 0.8040 |
| PC-AC | 0.133 ± 0.037 | 0.285 ± 0.066 | 3.6084 | 0.0671 | 0.0539 | 0.8181 | 0.0042 | 0.9485 |
| Thal-FOrb | 0.133 ± 0.085 | -0.072 ± 0.083 | 3.1366 | 0.0867 | 0.6285 | 0.4341 | 0.9820 | 0.3296 |
| PAG-LC | -0.041 ± 0.092 | 0.195 ± 0.074 | 3.0611 | 0.0904 | 0.0028 | 0.9579 | 0.0639 | 0.8022 |
| Amyg-Accu | 0.057 ± 0.033 | 0.136 ± 0.025 | 2.7236 | 0.1093 | 0.0535 | 0.8186 | 0.3071 | 0.5836 |
| PBN-Thal | 0.347 ± 0.104 | 0.074 ± 0.108 | 2.7213 | 0.1095 | 0.0026 | 0.9597 | 0.4310 | 0.5165 |
| FOrb-LC | -0.106 ± 0.103 | 0.188 ± 0.099 | 2.7095 | 0.1102 | 2.2387 | 0.1450 | 2.0467 | 0.1629 |
| VTA-Hipp | 0.098 ± 0.095 | 0.291 ± 0.108 | 2.6198 | 0.1160 | 2.3777 | 0.1336 | 0.4656 | 0.5002 |
| Amyg-Hypo | -0.021 ± 0.042 | 0.170 ± 0.109 | 2.4501 | 0.1280 | 0.2285 | 0.6361 | 1.2327 | 0.2757 |
| Amyg-IC | 0.275 ± 0.072 | 0.459 ± 0.073 | 2.3765 | 0.1337 | 0.2954 | 0.5908 | 0.8635 | 0.3602 |
| Thal-PAG | 0.099 ± 0.120 | 0.307 ± 0.110 | 1.8453 | 0.1845 | 0.7303 | 0.3996 | 0.7196 | 0.4030 |
| PC-Hipp | 0.051 ± 0.037 | -0.044 ± 0.062 | 1.7828 | 0.1918 | 0.0545 | 0.8171 | 0.5724 | 0.4552 |
| Amyg-Thal | 0.339 ± 0.239 | -0.147 ± 0.203 | 1.7596 | 0.1947 | 0.1175 | 0.7342 | 1.0351 | 0.3171 |
| LC-Thal | 0.311 ± 0.085 | 0.547 ± 0.113 | 1.7392 | 0.1972 | 1.6266 | 0.2120 | 0.0066 | 0.9360 |
| Hypo-FOrb | -0.070 ± 0.058 | 0.087 ± 0.100 | 1.4582 | 0.2366 | 0.2293 | 0.6355 | 0.3402 | 0.5641 |
| Accu-VTA | 0.036 ± 0.108 | 0.252 ± 0.054 | 1.4433 | 0.2390 | 4.8275 | 0.0359 | 2.3993 | 0.1319 |
| Thal-Hypo | -0.040 ± 0.165 | 0.274 ± 0.205 | 1.3324 | 0.2575 | 0.0347 | 0.8534 | 0.2960 | 0.5904 |
| Amyg-LC | -0.053 ± 0.074 | -0.156 ± 0.109 | 1.3122 | 0.2610 | 2.7180 | 0.1097 | 0.9457 | 0.3386 |
| int2-LC | -0.014 ± 0.113 | 0.153 ± 0.075 | 1.2955 | 0.2640 | 0.0904 | 0.7657 | 0.2864 | 0.5965 |
| AC-Amyg | 0.071 ± 0.156 | 0.146 ± 0.137 | 1.1979 | 0.2825 | 10.7624 | 0.0026 | 0.0120 | 0.9134 |
| Amyg-PAG | 0.071 ± 0.049 | 0.144 ± 0.055 | 1.0360 | 0.3169 | 0.2402 | 0.6276 | 0.1287 | 0.7223 |
| Thal-PC | 0.326 ± 0.094 | 0.144 ± 0.113 | 0.9459 | 0.3386 | 0.6847 | 0.4145 | 0.0007 | 0.9784 |
| Amyg-VTA | 0.127 ± 0.109 | 0.243 ± 0.081 | 0.6859 | 0.4141 | 0.1213 | 0.7301 | 0.2610 | 0.6132 |
| PBN-LC | 0.016 ± 0.131 | -0.085 ± 0.115 | 0.6377 | 0.4308 | 1.4567 | 0.2369 | 0.5435 | 0.4667 |
| Thal-HG | -0.161 ± 0.086 | -0.285 ± 0.078 | 0.5836 | 0.4509 | 0.8757 | 0.3569 | 0.0021 | 0.9641 |
| Amyg-Hipp | 0.046 ± 0.224 | -0.089 ± 0.194 | 0.5464 | 0.4655 | 2.0096 | 0.1666 | 1.3021 | 0.2628 |
| Thal-IC | 0.214 ± 0.123 | 0.144 ± 0.093 | 0.5159 | 0.4781 | 1.9440 | 0.1735 | 0.1613 | 0.6908 |
| Hypo-LC | -0.330 ± 0.087 | -0.214 ± 0.095 | 0.4768 | 0.4952 | 0.4198 | 0.5219 | 0.0659 | 0.7992 |
| Thal-Hipp | 0.165 ± 0.115 | 0.296 ± 0.122 | 0.4755 | 0.4958 | 0.2629 | 0.6119 | 8.1622 | 0.0077 |
| IC-Amyg | -0.270 ± 0.099 | -0.138 ± 0.115 | 0.4437 | 0.5104 | 0.4828 | 0.4925 | 1.0584 | 0.3118 |
| LC-Hypo | 0.040 ± 0.114 | 0.164 ± 0.098 | 0.4334 | 0.5153 | 0.4105 | 0.5266 | 4.9386 | 0.0339 |
| FOrb-Amyg | 0.053 ± 0.077 | -0.019 ± 0.056 | 0.4216 | 0.5211 | 0.0328 | 0.8575 | 1.6112 | 0.2141 |
| LC-PBN | -0.059 ± 0.054 | 0.020 ± 0.073 | 0.3852 | 0.5395 | 0.9332 | 0.3418 | 0.4837 | 0.4921 |
| Hipp-Accu | 0.372 ± 0.118 | 0.238 ± 0.132 | 0.3517 | 0.5576 | 0.1952 | 0.6618 | 0.0028 | 0.9580 |
| FOrb-Hypo | 0.197 ± 0.075 | 0.286 ± 0.128 | 0.3342 | 0.5675 | 0.0001 | 0.9922 | 0.5187 | 0.4770 |
| int1-IC | -0.050 ± 0.122 | -0.149 ± 0.151 | 0.3136 | 0.5796 | 0.1482 | 0.7030 | 0.0449 | 0.8337 |
| Thal-AC | 0.276 ± 0.073 | 0.175 ± 0.098 | 0.3131 | 0.5799 | 1.5010 | 0.2301 | 2.6289 | 0.1154 |
| Hypo-PBN | -0.033 ± 0.025 | -0.017 ± 0.029 | 0.3015 | 0.5870 | 0.6585 | 0.4235 | 0.0372 | 0.8484 |
| HG-IC | 0.119 ± 0.068 | 0.048 ± 0.053 | 0.3009 | 0.5874 | 0.7071 | 0.4070 | 0.2109 | 0.6494 |
| Hypo-Thal | 0.094 ± 0.032 | 0.113 ± 0.060 | 0.2980 | 0.5891 | 1.3482 | 0.2547 | 1.8843 | 0.1800 |
| Hipp-Amyg | -0.001 ± 0.139 | -0.135 ± 0.194 | 0.2381 | 0.6291 | 0.0481 | 0.8279 | 0.9861 | 0.3286 |
| Hypo-PAG | 0.245 ± 0.073 | 0.257 ± 0.094 | 0.1527 | 0.6988 | 1.7317 | 0.1982 | 1.5361 | 0.2248 |
| FOrb-Thal | 0.192 ± 0.074 | 0.171 ± 0.076 | 0.0957 | 0.7592 | 0.3338 | 0.5677 | 0.7913 | 0.3808 |
| VTA-Accu | -0.079 ± 0.138 | -0.009 ± 0.075 | 0.0884 | 0.7683 | 0.1527 | 0.6987 | 0.0035 | 0.9533 |
| int0-FOrb | 0.022 ± 0.068 | 0.065 ± 0.090 | 0.0728 | 0.7891 | 0.1735 | 0.6800 | 0.0441 | 0.8352 |
| LC-Hipp | 0.131 ± 0.107 | 0.170 ± 0.096 | 0.0708 | 0.7919 | 0.0045 | 0.9470 | 2.1490 | 0.1531 |
| PAG-PBN | 0.053 ± 0.068 | 0.077 ± 0.042 | 0.0681 | 0.7959 | 0.0000 | 0.9998 | 0.0305 | 0.8626 |
| Thal-Accu | -0.114 ± 0.075 | -0.161 ± 0.068 | 0.0595 | 0.8089 | 0.5984 | 0.4452 | 0.0586 | 0.8104 |
| AC-FOrb | -0.253 ± 0.067 | -0.239 ± 0.124 | 0.0227 | 0.8812 | 0.0555 | 0.8154 | 0.5380 | 0.4689 |
| IC-HG | 0.340 ± 0.100 | 0.318 ± 0.132 | 0.0110 | 0.9173 | 0.0182 | 0.8936 | 3.8692 | 0.0585 |
| IC-AC | 0.679 ± 0.155 | 0.732 ± 0.185 | 0.0072 | 0.9328 | 1.8775 | 0.1808 | 2.2378 | 0.1451 |
| FOrb-AC | -0.056 ± 0.119 | -0.032 ± 0.185 | 0.0019 | 0.9660 | 0.0696 | 0.7938 | 0.3179 | 0.5771 |
| PBN-Hypo | 0.102 ± 0.120 | 0.113 ± 0.148 | 0.0006 | 0.9809 | 0.1267 | 0.7244 | 0.7335 | 0.3986 |

**Table S2.2:** Variations of connectivity (DB) values in relation to group (FM vs HC) and STAI-Y-1 scores (2-way ANCOVA)

| **Connection** | **Group 1**  **DB values** | **Group 2**  **DB values** | **Main effect of Group** | | **Main effect of**  **STAI-Y-1 score** | | **Interaction Effect** | |
| --- | --- | --- | --- | --- | --- | --- | --- | --- |
|  | **(mean ± s.d.)** | **(mean ± s.d.)** | **F** | **p** | **F** | **p** | **F** | **p** |
| AC-PC | 0.402 ± 0.069 | 0.679 ± 0.066 | 8.6314 | 0.0063 | 1.8713 | 0.1815 | 0.1870 | 0.6685 |
| PC-AC | 0.133 ± 0.037 | 0.285 ± 0.066 | 5.5749 | 0.0249 | 2.8478 | 0.1019 | 1.5019 | 0.2299 |
| PC-Thal | 0.025 ± 0.031 | -0.089 ± 0.035 | 4.9352 | 0.0340 | 0.0922 | 0.7635 | 0.0190 | 0.8913 |
| FOrb-LC | -0.106 ± 0.103 | 0.188 ± 0.099 | 4.9031 | 0.0346 | 2.8665 | 0.1008 | 0.2210 | 0.6417 |
| Accu-VTA | 0.036 ± 0.108 | 0.252 ± 0.054 | 3.8175 | 0.0601 | 1.5690 | 0.2200 | 8.4893 | 0.0067 |
| PAG-LC | -0.041 ± 0.092 | 0.195 ± 0.074 | 3.7023 | 0.0639 | 0.6420 | 0.4293 | 0.1375 | 0.7134 |
| Amyg-Accu | 0.057 ± 0.033 | 0.136 ± 0.025 | 3.6441 | 0.0659 | 1.1699 | 0.2880 | 0.1074 | 0.7454 |
| Amyg-IC | 0.275 ± 0.072 | 0.459 ± 0.073 | 2.9276 | 0.0974 | 0.0920 | 0.7637 | 0.4280 | 0.5180 |
| PBN-Thal | 0.347 ± 0.104 | 0.074 ± 0.108 | 2.6435 | 0.1144 | 3.4869 | 0.0717 | 5.2230 | 0.0295 |
| VTA-Hipp | 0.098 ± 0.095 | 0.291 ± 0.108 | 2.5565 | 0.1203 | 3.0369 | 0.0916 | 2.1602 | 0.1520 |
| AC-IC | 0.029 ± 0.029 | -0.042 ± 0.022 | 2.5207 | 0.1228 | 2.2863 | 0.1410 | 0.0022 | 0.9629 |
| LC-Thal | 0.311 ± 0.085 | 0.547 ± 0.113 | 2.3249 | 0.1378 | 0.2567 | 0.6161 | 0.5266 | 0.4737 |
| Amyg-Hypo | -0.021 ± 0.042 | 0.170 ± 0.109 | 2.2572 | 0.1434 | 2.2796 | 0.1415 | 0.3056 | 0.5845 |
| Thal-Hypo | -0.040 ± 0.165 | 0.274 ± 0.205 | 2.1527 | 0.1527 | 3.0022 | 0.0934 | 2.0523 | 0.1623 |
| Thal-FOrb | 0.133 ± 0.085 | -0.072 ± 0.083 | 1.9232 | 0.1757 | 4.3302 | 0.0461 | 0.1514 | 0.6999 |
| Hypo-FOrb | -0.070 ± 0.058 | 0.087 ± 0.100 | 1.7417 | 0.1969 | 0.5071 | 0.4819 | 4.4854 | 0.0426 |
| Amyg-Thal | 0.339 ± 0.239 | -0.147 ± 0.203 | 1.7255 | 0.1989 | 0.6110 | 0.4406 | 0.8390 | 0.3670 |
| Hypo-LC | -0.330 ± 0.087 | -0.214 ± 0.095 | 1.5005 | 0.2301 | 4.8204 | 0.0360 | 0.1002 | 0.7538 |
| Thal-PAG | 0.099 ± 0.120 | 0.307 ± 0.110 | 1.4057 | 0.2451 | 0.0153 | 0.9023 | 0.3971 | 0.5334 |
| Thal-PC | 0.326 ± 0.094 | 0.144 ± 0.113 | 1.2951 | 0.2641 | 0.0383 | 0.8462 | 0.4631 | 0.5014 |
| PC-Hipp | 0.051 ± 0.037 | -0.044 ± 0.062 | 1.2538 | 0.2717 | 2.9257 | 0.0975 | 0.9920 | 0.3272 |
| Thal-HG | -0.161 ± 0.086 | -0.285 ± 0.078 | 1.2134 | 0.2794 | 0.6290 | 0.4340 | 0.4232 | 0.5203 |
| HG-IC | 0.119 ± 0.068 | 0.048 ± 0.053 | 1.1306 | 0.2961 | 3.8099 | 0.0603 | 0.0396 | 0.8436 |
| int2-LC | -0.014 ± 0.113 | 0.153 ± 0.075 | 1.0442 | 0.3150 | 0.3334 | 0.5680 | 1.1247 | 0.2974 |
| Amyg-PAG | 0.071 ± 0.049 | 0.144 ± 0.055 | 0.8669 | 0.3593 | 0.0051 | 0.9435 | 0.2069 | 0.6525 |
| IC-Amyg | -0.270 ± 0.099 | -0.138 ± 0.115 | 0.7433 | 0.3954 | 0.1218 | 0.7296 | 0.0008 | 0.9777 |
| Amyg-VTA | 0.127 ± 0.109 | 0.243 ± 0.081 | 0.6973 | 0.4103 | 0.2543 | 0.6178 | 0.0469 | 0.8300 |
| LC-PBN | -0.059 ± 0.054 | 0.020 ± 0.073 | 0.6458 | 0.4279 | 0.0288 | 0.8663 | 0.2314 | 0.6340 |
| LC-Hypo | 0.040 ± 0.114 | 0.164 ± 0.098 | 0.4761 | 0.4955 | 0.1629 | 0.6894 | 0.0668 | 0.7978 |
| Thal-AC | 0.276 ± 0.073 | 0.175 ± 0.098 | 0.4712 | 0.4977 | 0.5495 | 0.4643 | 0.0244 | 0.8770 |
| Amyg-LC | -0.053 ± 0.074 | -0.156 ± 0.109 | 0.4658 | 0.5001 | 0.2914 | 0.5933 | 0.6147 | 0.4392 |
| FOrb-Amyg | 0.053 ± 0.077 | -0.019 ± 0.056 | 0.4294 | 0.5173 | 0.0465 | 0.8307 | 0.5433 | 0.4668 |
| Hipp-Accu | 0.372 ± 0.118 | 0.238 ± 0.132 | 0.3844 | 0.5399 | 0.3071 | 0.5836 | 0.0232 | 0.8799 |
| Thal-Hipp | 0.165 ± 0.115 | 0.296 ± 0.122 | 0.3724 | 0.5463 | 0.7805 | 0.3840 | 0.9969 | 0.3260 |
| FOrb-Hypo | 0.197 ± 0.075 | 0.286 ± 0.128 | 0.3115 | 0.5809 | 0.0431 | 0.8370 | 0.4862 | 0.4910 |
| int1-IC | -0.050 ± 0.122 | -0.149 ± 0.151 | 0.3100 | 0.5818 | 0.2184 | 0.6436 | 0.7524 | 0.3926 |
| Hypo-PBN | -0.033 ± 0.025 | -0.017 ± 0.029 | 0.2810 | 0.5999 | 0.9633 | 0.3342 | 1.4242 | 0.2421 |
| Thal-Accu | -0.114 ± 0.075 | -0.161 ± 0.068 | 0.2280 | 0.6365 | 0.1606 | 0.6915 | 0.4051 | 0.5293 |
| PBN-LC | 0.016 ± 0.131 | -0.085 ± 0.115 | 0.2050 | 0.6540 | 0.2738 | 0.6046 | 0.3340 | 0.5676 |
| VTA-Accu | -0.079 ± 0.138 | -0.009 ± 0.075 | 0.1623 | 0.6899 | 0.0080 | 0.9295 | 0.3726 | 0.5462 |
| Hipp-Amyg | -0.001 ± 0.139 | -0.135 ± 0.194 | 0.1334 | 0.7175 | 2.8436 | 0.1021 | 6.5041 | 0.0161 |
| Thal-IC | 0.214 ± 0.123 | 0.144 ± 0.093 | 0.1325 | 0.7184 | 0.0777 | 0.7824 | 0.0043 | 0.9481 |
| Hypo-Thal | 0.094 ± 0.032 | 0.113 ± 0.060 | 0.1230 | 0.7282 | 0.2194 | 0.6429 | 0.1379 | 0.7130 |
| LC-Hipp | 0.131 ± 0.107 | 0.170 ± 0.096 | 0.1000 | 0.7540 | 0.2021 | 0.6563 | 0.8176 | 0.3731 |
| IC-AC | 0.679 ± 0.155 | 0.732 ± 0.185 | 0.0763 | 0.7843 | 0.2298 | 0.6351 | 0.0811 | 0.7778 |
| PAG-PBN | 0.053 ± 0.068 | 0.077 ± 0.042 | 0.0650 | 0.8005 | 0.0083 | 0.9280 | 0.8507 | 0.3637 |
| AC-Amyg | 0.071 ± 0.156 | 0.146 ± 0.137 | 0.0537 | 0.8183 | 0.4418 | 0.5113 | 0.1257 | 0.7255 |
| Amyg-Hipp | 0.046 ± 0.224 | -0.089 ± 0.194 | 0.0281 | 0.8681 | 3.4516 | 0.0730 | 0.6767 | 0.4172 |
| IC-HG | 0.340 ± 0.100 | 0.318 ± 0.132 | 0.0122 | 0.9129 | 0.0204 | 0.8873 | 1.4656 | 0.2355 |
| AC-FOrb | -0.253 ± 0.067 | -0.239 ± 0.124 | 0.0071 | 0.9333 | 0.0111 | 0.9166 | 0.1850 | 0.6702 |
| PBN-Hypo | 0.102 ± 0.120 | 0.113 ± 0.148 | 0.0068 | 0.9347 | 0.8815 | 0.3553 | 0.1331 | 0.7178 |
| int0-FOrb | 0.022 ± 0.068 | 0.065 ± 0.090 | 0.0049 | 0.9444 | 5.0311 | 0.0324 | 0.7519 | 0.3928 |
| FOrb-AC | -0.056 ± 0.119 | -0.032 ± 0.185 | 0.0046 | 0.9465 | 0.0576 | 0.8119 | 0.6388 | 0.4304 |
| FOrb-Thal | 0.192 ± 0.074 | 0.171 ± 0.076 | 0.0043 | 0.9480 | 3.0948 | 0.0887 | 0.3091 | 0.5824 |
| Hypo-PAG | 0.245 ± 0.073 | 0.257 ± 0.094 | 0.0001 | 0.9930 | 0.3817 | 0.5413 | 0.2565 | 0.6162 |

**Table S2.3:** Variations of connectivity (DB) values in relation to group (FM vs HC) and STAI-Y-2 scores (2-way ANCOVA)

| **Connection** | **Group 1**  **DB values** | **Group 2**  **DB values** | **Main effect of Group** | | **Main effect of**  **STAI-Y-2 score** | | **Interaction Effect** | |
| --- | --- | --- | --- | --- | --- | --- | --- | --- |
|  | **(mean ± s.d.)** | **(mean ± s.d.)** | **F** | **p** | **F** | **p** | **F** | **p** |
| AC-PC | 0.402 ± 0.069 | 0.679 ± 0.066 | 7.0914 | 0.0125 | 0.7008 | 0.4094 | 0.3864 | 0.5391 |
| PC-AC | 0.133 ± 0.037 | 0.285 ± 0.066 | 6.4954 | 0.0164 | 2.2112 | 0.1478 | 1.0001 | 0.3256 |
| FOrb-LC | -0.106 ± 0.103 | 0.188 ± 0.099 | 4.3273 | 0.0464 | 3.9806 | 0.0555 | 0.5670 | 0.4575 |
| PC-Thal | 0.025 ± 0.031 | -0.089 ± 0.035 | 4.1350 | 0.0512 | 1.7327 | 0.1984 | 2.1207 | 0.1561 |
| Amyg-Accu | 0.057 ± 0.033 | 0.136 ± 0.025 | 3.7197 | 0.0636 | 1.6830 | 0.2048 | 0.0009 | 0.9758 |
| PAG-LC | -0.041 ± 0.092 | 0.195 ± 0.074 | 3.4014 | 0.0754 | 0.5796 | 0.4526 | 0.4304 | 0.5170 |
| Accu-VTA | 0.036 ± 0.108 | 0.252 ± 0.054 | 2.9760 | 0.0952 | 1.1355 | 0.2954 | 8.5372 | 0.0067 |
| LC-Thal | 0.311 ± 0.085 | 0.547 ± 0.113 | 2.8033 | 0.1048 | 1.2682 | 0.2693 | 2.0272 | 0.1652 |
| VTA-Hipp | 0.098 ± 0.095 | 0.291 ± 0.108 | 2.6292 | 0.1157 | 2.1421 | 0.1541 | 0.4148 | 0.5246 |
| PBN-Thal | 0.347 ± 0.104 | 0.074 ± 0.108 | 2.3952 | 0.1326 | 1.7513 | 0.1961 | 5.7663 | 0.0230 |
| Thal-Hypo | -0.040 ± 0.165 | 0.274 ± 0.205 | 2.3133 | 0.1391 | 4.2088 | 0.0493 | 2.8755 | 0.1007 |
| Hypo-LC | -0.330 ± 0.087 | -0.214 ± 0.095 | 2.2649 | 0.1432 | 7.2345 | 0.0117 | 0.0092 | 0.9241 |
| Amyg-IC | 0.275 ± 0.072 | 0.459 ± 0.073 | 2.2564 | 0.1439 | 0.0383 | 0.8461 | 0.0206 | 0.8870 |
| Thal-HG | -0.161 ± 0.086 | -0.285 ± 0.078 | 2.1202 | 0.1561 | 0.3290 | 0.5706 | 0.0866 | 0.7707 |
| AC-IC | 0.029 ± 0.029 | -0.042 ± 0.022 | 1.7427 | 0.1971 | 0.0950 | 0.7601 | 0.0006 | 0.9806 |
| HG-IC | 0.119 ± 0.068 | 0.048 ± 0.053 | 1.5761 | 0.2194 | 5.4856 | 0.0262 | 0.0087 | 0.9263 |
| Hypo-FOrb | -0.070 ± 0.058 | 0.087 ± 0.100 | 1.4677 | 0.2355 | 0.6575 | 0.4241 | 7.1072 | 0.0124 |
| Thal-PC | 0.326 ± 0.094 | 0.144 ± 0.113 | 1.2870 | 0.2659 | 0.4409 | 0.5119 | 0.1531 | 0.6984 |
| Amyg-Hypo | -0.021 ± 0.042 | 0.170 ± 0.109 | 1.1203 | 0.2986 | 2.2585 | 0.1437 | 0.1890 | 0.6670 |
| IC-Amyg | -0.270 ± 0.099 | -0.138 ± 0.115 | 1.1120 | 0.3004 | 0.6661 | 0.4211 | 0.2438 | 0.6252 |
| Thal-PAG | 0.099 ± 0.120 | 0.307 ± 0.110 | 1.0379 | 0.3167 | 0.0674 | 0.7970 | 0.8450 | 0.3656 |
| int2-LC | -0.014 ± 0.113 | 0.153 ± 0.075 | 1.0065 | 0.3240 | 1.8470 | 0.1846 | 5.6018 | 0.0248 |
| PC-Hipp | 0.051 ± 0.037 | -0.044 ± 0.062 | 0.9509 | 0.3376 | 2.3484 | 0.1362 | 1.6420 | 0.2102 |
| Amyg-Thal | 0.339 ± 0.239 | -0.147 ± 0.203 | 0.9453 | 0.3390 | 6.1379 | 0.0193 | 0.4751 | 0.4961 |
| Thal-FOrb | 0.133 ± 0.085 | -0.072 ± 0.083 | 0.8794 | 0.3561 | 11.5639 | 0.0020 | 0.5287 | 0.4730 |
| Thal-Accu | -0.114 ± 0.075 | -0.161 ± 0.068 | 0.7495 | 0.3937 | 0.0036 | 0.9527 | 0.0781 | 0.7819 |
| LC-Hypo | 0.040 ± 0.114 | 0.164 ± 0.098 | 0.6793 | 0.4165 | 1.0848 | 0.3062 | 2.3789 | 0.1338 |
| int1-IC | -0.050 ± 0.122 | -0.149 ± 0.151 | 0.6097 | 0.4412 | 0.0300 | 0.8637 | 0.6438 | 0.4289 |
| Amyg-VTA | 0.127 ± 0.109 | 0.243 ± 0.081 | 0.5695 | 0.4565 | 0.0350 | 0.8530 | 0.0003 | 0.9854 |
| Thal-AC | 0.276 ± 0.073 | 0.175 ± 0.098 | 0.4601 | 0.5029 | 0.9209 | 0.3452 | 0.0326 | 0.8579 |
| Thal-Hipp | 0.165 ± 0.115 | 0.296 ± 0.122 | 0.4297 | 0.5173 | 0.8243 | 0.3714 | 0.0119 | 0.9138 |
| Amyg-LC | -0.053 ± 0.074 | -0.156 ± 0.109 | 0.3710 | 0.5472 | 1.6353 | 0.2111 | 2.2783 | 0.1420 |
| Amyg-PAG | 0.071 ± 0.049 | 0.144 ± 0.055 | 0.3572 | 0.5547 | 0.0742 | 0.7872 | 2.1769 | 0.1509 |
| Hipp-Accu | 0.372 ± 0.118 | 0.238 ± 0.132 | 0.3194 | 0.5763 | 0.5272 | 0.4736 | 0.0099 | 0.9215 |
| Thal-IC | 0.214 ± 0.123 | 0.144 ± 0.093 | 0.2678 | 0.6088 | 0.0016 | 0.9686 | 0.0093 | 0.9239 |
| FOrb-Amyg | 0.053 ± 0.077 | -0.019 ± 0.056 | 0.2665 | 0.6096 | 0.0311 | 0.8612 | 0.4993 | 0.4854 |
| FOrb-Hypo | 0.197 ± 0.075 | 0.286 ± 0.128 | 0.2390 | 0.6286 | 0.0489 | 0.8265 | 2.5534 | 0.1209 |
| Hypo-PBN | -0.033 ± 0.025 | -0.017 ± 0.029 | 0.2160 | 0.6456 | 1.0900 | 0.3051 | 2.3083 | 0.1395 |
| Hypo-Thal | 0.094 ± 0.032 | 0.113 ± 0.060 | 0.1926 | 0.6640 | 0.0840 | 0.7740 | 0.5693 | 0.4566 |
| FOrb-AC | -0.056 ± 0.119 | -0.032 ± 0.185 | 0.1503 | 0.7011 | 0.7113 | 0.4059 | 0.1928 | 0.6639 |
| AC-FOrb | -0.253 ± 0.067 | -0.239 ± 0.124 | 0.1392 | 0.7118 | 0.2708 | 0.6067 | 0.1348 | 0.7161 |
| PBN-Hypo | 0.102 ± 0.120 | 0.113 ± 0.148 | 0.1361 | 0.7149 | 1.6169 | 0.2136 | 0.0486 | 0.8271 |
| LC-PBN | -0.059 ± 0.054 | 0.020 ± 0.073 | 0.1196 | 0.7319 | 0.1488 | 0.7025 | 2.7093 | 0.1106 |
| Hypo-PAG | 0.245 ± 0.073 | 0.257 ± 0.094 | 0.1156 | 0.7363 | 0.0509 | 0.8231 | 0.5800 | 0.4525 |
| IC-AC | 0.679 ± 0.155 | 0.732 ± 0.185 | 0.0889 | 0.7677 | 0.5544 | 0.4625 | 0.0946 | 0.7606 |
| FOrb-Thal | 0.192 ± 0.074 | 0.171 ± 0.076 | 0.0729 | 0.7890 | 4.0593 | 0.0533 | 1.0740 | 0.3086 |
| VTA-Accu | -0.079 ± 0.138 | -0.009 ± 0.075 | 0.0685 | 0.7954 | 0.4780 | 0.4948 | 0.0054 | 0.9417 |
| PAG-PBN | 0.053 ± 0.068 | 0.077 ± 0.042 | 0.0505 | 0.8237 | 0.1068 | 0.7462 | 0.5805 | 0.4523 |
| LC-Hipp | 0.131 ± 0.107 | 0.170 ± 0.096 | 0.0467 | 0.8304 | 0.1039 | 0.7496 | 0.2276 | 0.6369 |
| PBN-LC | 0.016 ± 0.131 | -0.085 ± 0.115 | 0.0310 | 0.8614 | 0.5482 | 0.4650 | 0.0206 | 0.8869 |
| int0-FOrb | 0.022 ± 0.068 | 0.065 ± 0.090 | 0.0153 | 0.9025 | 6.0852 | 0.0198 | 0.4216 | 0.5213 |
| Hipp-Amyg | -0.001 ± 0.139 | -0.135 ± 0.194 | 0.0023 | 0.9621 | 6.8129 | 0.0142 | 0.7559 | 0.3918 |
| IC-HG | 0.340 ± 0.100 | 0.318 ± 0.132 | 0.0008 | 0.9772 | 0.1345 | 0.7165 | 0.5551 | 0.4622 |
| Amyg-Hipp | 0.046 ± 0.224 | -0.089 ± 0.194 | 0.0002 | 0.9878 | 2.8833 | 0.1002 | 0.0802 | 0.7790 |
| AC-Amyg | 0.071 ± 0.156 | 0.146 ± 0.137 | 0.0001 | 0.9937 | 1.6687 | 0.2066 | 0.8391 | 0.3672 |

**Table S2.4:** Variations of connectivity (DB) values in relation to group (FM vs HC) and PCS Total scores (2-way ANCOVA)

| **Connection** | **Group 1**  **DB values** | **Group 2**  **DB values** | **Main effect of Group** | | **Main effect of**  **PCS Total score** | | **Interaction Effect** | |
| --- | --- | --- | --- | --- | --- | --- | --- | --- |
|  | **(mean ± s.d.)** | **(mean ± s.d.)** | **F** | **p** | **F** | **p** | **F** | **p** |
| AC-PC | 0.402 ± 0.069 | 0.679 ± 0.066 | 6.7076 | 0.0153 | 0.4288 | 0.5181 | 0.0710 | 0.7920 |
| Amyg-Accu | 0.057 ± 0.033 | 0.136 ± 0.025 | 4.5603 | 0.0419 | 2.5274 | 0.1235 | 0.0297 | 0.8645 |
| PC-Thal | 0.025 ± 0.031 | -0.089 ± 0.035 | 4.3344 | 0.0470 | 0.1734 | 0.6804 | 2.9222 | 0.0988 |
| PAG-LC | -0.041 ± 0.092 | 0.195 ± 0.074 | 4.2041 | 0.0501 | 0.6757 | 0.4183 | 0.2302 | 0.6352 |
| FOrb-LC | -0.106 ± 0.103 | 0.188 ± 0.099 | 3.6300 | 0.0674 | 0.4518 | 0.5072 | 2.9967 | 0.0948 |
| Thal-PAG | 0.099 ± 0.120 | 0.307 ± 0.110 | 3.5200 | 0.0715 | 1.6016 | 0.2165 | 1.6213 | 0.2138 |
| Accu-VTA | 0.036 ± 0.108 | 0.252 ± 0.054 | 3.3198 | 0.0795 | 0.4497 | 0.5082 | 0.0706 | 0.7925 |
| Thal-HG | -0.161 ± 0.086 | -0.285 ± 0.078 | 2.6794 | 0.1133 | 2.7826 | 0.1069 | 0.0075 | 0.9316 |
| Amyg-Thal | 0.339 ± 0.239 | -0.147 ± 0.203 | 2.4282 | 0.1308 | 0.1394 | 0.7118 | 2.3044 | 0.1406 |
| Thal-FOrb | 0.133 ± 0.085 | -0.072 ± 0.083 | 1.9281 | 0.1763 | 0.2455 | 0.6243 | 0.0029 | 0.9574 |
| Amyg-IC | 0.275 ± 0.072 | 0.459 ± 0.073 | 1.9043 | 0.1789 | 0.2394 | 0.6286 | 4.1796 | 0.0508 |
| VTA-Hipp | 0.098 ± 0.095 | 0.291 ± 0.108 | 1.8407 | 0.1861 | 1.1415 | 0.2948 | 0.5334 | 0.4715 |
| LC-Thal | 0.311 ± 0.085 | 0.547 ± 0.113 | 1.5946 | 0.2175 | 0.0789 | 0.7810 | 0.6796 | 0.4169 |
| PC-AC | 0.133 ± 0.037 | 0.285 ± 0.066 | 1.5911 | 0.2180 | 0.7676 | 0.3887 | 0.7005 | 0.4100 |
| Thal-PC | 0.326 ± 0.094 | 0.144 ± 0.113 | 1.3584 | 0.2540 | 0.0004 | 0.9841 | 5.3650 | 0.0284 |
| Thal-Hypo | -0.040 ± 0.165 | 0.274 ± 0.205 | 1.1730 | 0.2884 | 0.0414 | 0.8402 | 0.3225 | 0.5748 |
| Amyg-PAG | 0.071 ± 0.049 | 0.144 ± 0.055 | 1.1708 | 0.2888 | 0.1065 | 0.7467 | 0.1116 | 0.7409 |
| PC-Hipp | 0.051 ± 0.037 | -0.044 ± 0.062 | 1.1458 | 0.2939 | 0.0635 | 0.8030 | 1.7930 | 0.1917 |
| Hypo-LC | -0.330 ± 0.087 | -0.214 ± 0.095 | 1.1258 | 0.2981 | 0.0252 | 0.8752 | 0.8273 | 0.3711 |
| int2-LC | -0.014 ± 0.113 | 0.153 ± 0.075 | 1.0949 | 0.3047 | 0.0908 | 0.7655 | 0.8168 | 0.3741 |
| Thal-Accu | -0.114 ± 0.075 | -0.161 ± 0.068 | 1.0126 | 0.3232 | 1.7862 | 0.1925 | 0.0028 | 0.9581 |
| int1-IC | -0.050 ± 0.122 | -0.149 ± 0.151 | 0.8897 | 0.3539 | 1.8407 | 0.1861 | 2.4917 | 0.1261 |
| Amyg-Hypo | -0.021 ± 0.042 | 0.170 ± 0.109 | 0.8855 | 0.3551 | 0.4317 | 0.5167 | 1.3783 | 0.2506 |
| FOrb-Amyg | 0.053 ± 0.077 | -0.019 ± 0.056 | 0.8658 | 0.3604 | 0.1566 | 0.6954 | 6.5551 | 0.0164 |
| PAG-PBN | 0.053 ± 0.068 | 0.077 ± 0.042 | 0.8607 | 0.3618 | 3.7543 | 0.0632 | 0.0070 | 0.9338 |
| Hypo-FOrb | -0.070 ± 0.058 | 0.087 ± 0.100 | 0.8591 | 0.3622 | 0.0146 | 0.9048 | 0.6178 | 0.4387 |
| PBN-Thal | 0.347 ± 0.104 | 0.074 ± 0.108 | 0.8034 | 0.3780 | 0.3964 | 0.5342 | 0.0099 | 0.9214 |
| HG-IC | 0.119 ± 0.068 | 0.048 ± 0.053 | 0.7964 | 0.3801 | 0.6679 | 0.4209 | 3.1774 | 0.0859 |
| AC-IC | 0.029 ± 0.029 | -0.042 ± 0.022 | 0.7469 | 0.3951 | 0.5196 | 0.4772 | 0.0165 | 0.8988 |
| VTA-Accu | -0.079 ± 0.138 | -0.009 ± 0.075 | 0.7438 | 0.3960 | 0.7923 | 0.3813 | 0.0898 | 0.7667 |
| Hipp-Accu | 0.372 ± 0.118 | 0.238 ± 0.132 | 0.7139 | 0.4056 | 0.3760 | 0.5449 | 0.0411 | 0.8410 |
| Thal-Hipp | 0.165 ± 0.115 | 0.296 ± 0.122 | 0.6893 | 0.4137 | 0.0031 | 0.9558 | 3.5231 | 0.0714 |
| Hipp-Amyg | -0.001 ± 0.139 | -0.135 ± 0.194 | 0.6793 | 0.4170 | 0.8679 | 0.3598 | 0.9301 | 0.3434 |
| PBN-LC | 0.016 ± 0.131 | -0.085 ± 0.115 | 0.6288 | 0.4347 | 0.7870 | 0.3828 | 0.1542 | 0.6976 |
| IC-Amyg | -0.270 ± 0.099 | -0.138 ± 0.115 | 0.5552 | 0.4627 | 0.0013 | 0.9716 | 0.5009 | 0.4852 |
| FOrb-Hypo | 0.197 ± 0.075 | 0.286 ± 0.128 | 0.5202 | 0.4769 | 0.1276 | 0.7237 | 0.3968 | 0.5341 |
| Thal-AC | 0.276 ± 0.073 | 0.175 ± 0.098 | 0.5103 | 0.4811 | 0.1678 | 0.6853 | 0.3529 | 0.5574 |
| Amyg-LC | -0.053 ± 0.074 | -0.156 ± 0.109 | 0.3610 | 0.5529 | 0.1325 | 0.7187 | 1.3392 | 0.2573 |
| int0-FOrb | 0.022 ± 0.068 | 0.065 ± 0.090 | 0.3489 | 0.5596 | 2.1330 | 0.1557 | 2.4787 | 0.1270 |
| LC-Hipp | 0.131 ± 0.107 | 0.170 ± 0.096 | 0.3150 | 0.5792 | 1.0728 | 0.3095 | 0.4948 | 0.4878 |
| FOrb-AC | -0.056 ± 0.119 | -0.032 ± 0.185 | 0.3149 | 0.5793 | 0.9571 | 0.3366 | 2.5694 | 0.1206 |
| AC-FOrb | -0.253 ± 0.067 | -0.239 ± 0.124 | 0.2637 | 0.6118 | 0.1684 | 0.6847 | 7.5113 | 0.0107 |
| PBN-Hypo | 0.102 ± 0.120 | 0.113 ± 0.148 | 0.2398 | 0.6283 | 0.4632 | 0.5019 | 0.0002 | 0.9890 |
| FOrb-Thal | 0.192 ± 0.074 | 0.171 ± 0.076 | 0.2308 | 0.6348 | 0.0003 | 0.9859 | 0.1104 | 0.7423 |
| Hypo-PBN | -0.033 ± 0.025 | -0.017 ± 0.029 | 0.1727 | 0.6810 | 0.0625 | 0.8044 | 3.5244 | 0.0713 |
| Amyg-VTA | 0.127 ± 0.109 | 0.243 ± 0.081 | 0.1569 | 0.6951 | 2.2521 | 0.1450 | 0.0061 | 0.9385 |
| LC-Hypo | 0.040 ± 0.114 | 0.164 ± 0.098 | 0.1232 | 0.7283 | 2.9354 | 0.0981 | 0.0542 | 0.8177 |
| Hypo-PAG | 0.245 ± 0.073 | 0.257 ± 0.094 | 0.0953 | 0.7600 | 0.1854 | 0.6702 | 1.5902 | 0.2181 |
| IC-HG | 0.340 ± 0.100 | 0.318 ± 0.132 | 0.0462 | 0.8314 | 0.0909 | 0.7653 | 0.7598 | 0.3911 |
| LC-PBN | -0.059 ± 0.054 | 0.020 ± 0.073 | 0.0426 | 0.8379 | 0.5226 | 0.4759 | 3.6916 | 0.0653 |
| Amyg-Hipp | 0.046 ± 0.224 | -0.089 ± 0.194 | 0.0391 | 0.8447 | 0.0000 | 0.9978 | 0.1189 | 0.7329 |
| AC-Amyg | 0.071 ± 0.156 | 0.146 ± 0.137 | 0.0255 | 0.8742 | 0.0376 | 0.8476 | 0.2355 | 0.6314 |
| Thal-IC | 0.214 ± 0.123 | 0.144 ± 0.093 | 0.0152 | 0.9028 | 0.1206 | 0.7310 | 0.3620 | 0.5524 |
| Hypo-Thal | 0.094 ± 0.032 | 0.113 ± 0.060 | 0.0026 | 0.9601 | 0.2571 | 0.6163 | 0.1573 | 0.6948 |
| IC-AC | 0.679 ± 0.155 | 0.732 ± 0.185 | 0.0002 | 0.9883 | 0.0106 | 0.9187 | 0.7237 | 0.4024 |

**Table S2.5:** Variations of connectivity (DB) values in relation to group (FM vs HC) and scores on Helplessness domain of PCS (2-way ANCOVA)

| **Connection** | **Group 1**  **DB values** | **Group 2**  **DB values** | **Main effect of Group** | | **Main effect of**  **PCS Helplessness** | | **Interaction Effect** | |
| --- | --- | --- | --- | --- | --- | --- | --- | --- |
|  | **(mean ± s.d.)** | **(mean ± s.d.)** | **F** | **p** | **F** | **p** | **F** | **p** |
| AC-PC | 0.402 ± 0.069 | 0.679 ± 0.066 | 7.2188 | 0.0122 | 0.6724 | 0.4194 | 0.0085 | 0.9274 |
| Accu-VTA | 0.036 ± 0.108 | 0.252 ± 0.054 | 4.8998 | 0.0355 | 1.6991 | 0.2034 | 0.2418 | 0.6269 |
| Amyg-Accu | 0.057 ± 0.033 | 0.136 ± 0.025 | 4.7176 | 0.0388 | 2.7082 | 0.1114 | 0.0115 | 0.9153 |
| FOrb-LC | -0.106 ± 0.103 | 0.188 ± 0.099 | 4.0237 | 0.0550 | 0.7765 | 0.3860 | 2.0649 | 0.1622 |
| PC-Thal | 0.025 ± 0.031 | -0.089 ± 0.035 | 3.2943 | 0.0806 | 0.7477 | 0.3948 | 2.1444 | 0.1546 |
| Thal-PAG | 0.099 ± 0.120 | 0.307 ± 0.110 | 3.2485 | 0.0827 | 1.3706 | 0.2519 | 0.7546 | 0.3927 |
| PAG-LC | -0.041 ± 0.092 | 0.195 ± 0.074 | 3.1611 | 0.0867 | 0.1248 | 0.7266 | 0.5269 | 0.4742 |
| Amyg-IC | 0.275 ± 0.072 | 0.459 ± 0.073 | 2.2094 | 0.1488 | 0.3940 | 0.5355 | 5.7439 | 0.0237 |
| Thal-HG | -0.161 ± 0.086 | -0.285 ± 0.078 | 2.1736 | 0.1520 | 1.9274 | 0.1764 | 0.1631 | 0.6895 |
| LC-Thal | 0.311 ± 0.085 | 0.547 ± 0.113 | 2.1494 | 0.1542 | 0.3467 | 0.5609 | 2.0329 | 0.1654 |
| int2-LC | -0.014 ± 0.113 | 0.153 ± 0.075 | 2.1121 | 0.1577 | 1.0029 | 0.3255 | 0.8054 | 0.3774 |
| Thal-FOrb | 0.133 ± 0.085 | -0.072 ± 0.083 | 2.0649 | 0.1622 | 0.1745 | 0.6794 | 0.4057 | 0.5295 |
| PC-Hipp | 0.051 ± 0.037 | -0.044 ± 0.062 | 1.9979 | 0.1689 | 0.6944 | 0.4120 | 2.2478 | 0.1454 |
| VTA-Hipp | 0.098 ± 0.095 | 0.291 ± 0.108 | 1.7545 | 0.1964 | 1.0241 | 0.3205 | 0.5238 | 0.4755 |
| PAG-PBN | 0.053 ± 0.068 | 0.077 ± 0.042 | 1.6914 | 0.2044 | 6.7733 | 0.0148 | 0.0000 | 0.9971 |
| Thal-PC | 0.326 ± 0.094 | 0.144 ± 0.113 | 1.6639 | 0.2080 | 0.0947 | 0.7606 | 3.3427 | 0.0786 |
| Amyg-Thal | 0.339 ± 0.239 | -0.147 ± 0.203 | 1.6036 | 0.2162 | 0.0053 | 0.9423 | 0.7770 | 0.3859 |
| PBN-Thal | 0.347 ± 0.104 | 0.074 ± 0.108 | 1.5997 | 0.2168 | 0.0004 | 0.9833 | 0.7131 | 0.4058 |
| FOrb-Amyg | 0.053 ± 0.077 | -0.019 ± 0.056 | 1.5590 | 0.2225 | 0.9802 | 0.3309 | 4.2338 | 0.0494 |
| PC-AC | 0.133 ± 0.037 | 0.285 ± 0.066 | 1.3716 | 0.2518 | 1.0080 | 0.3243 | 0.0858 | 0.7719 |
| Amyg-PAG | 0.071 ± 0.049 | 0.144 ± 0.055 | 1.2756 | 0.2687 | 0.1683 | 0.6849 | 0.1342 | 0.7169 |
| Hypo-LC | -0.330 ± 0.087 | -0.214 ± 0.095 | 1.2491 | 0.2736 | 0.0440 | 0.8354 | 2.5360 | 0.1229 |
| Hipp-Amyg | -0.001 ± 0.139 | -0.135 ± 0.194 | 1.2390 | 0.2755 | 2.0683 | 0.1619 | 0.0775 | 0.7828 |
| int1-IC | -0.050 ± 0.122 | -0.149 ± 0.151 | 1.0686 | 0.3104 | 2.2503 | 0.1452 | 4.2634 | 0.0487 |
| Hypo-FOrb | -0.070 ± 0.058 | 0.087 ± 0.100 | 1.0085 | 0.3242 | 0.0124 | 0.9121 | 4.9433 | 0.0348 |
| Thal-Hipp | 0.165 ± 0.115 | 0.296 ± 0.122 | 0.9885 | 0.3289 | 0.0678 | 0.7966 | 2.6289 | 0.1166 |
| HG-IC | 0.119 ± 0.068 | 0.048 ± 0.053 | 0.9102 | 0.3485 | 0.8311 | 0.3700 | 4.0384 | 0.0546 |
| VTA-Accu | -0.079 ± 0.138 | -0.009 ± 0.075 | 0.8571 | 0.3628 | 0.9959 | 0.3272 | 0.0235 | 0.8794 |
| Amyg-Hypo | -0.021 ± 0.042 | 0.170 ± 0.109 | 0.8332 | 0.3694 | 0.5424 | 0.4678 | 2.2273 | 0.1472 |
| Thal-Accu | -0.114 ± 0.075 | -0.161 ± 0.068 | 0.7236 | 0.4024 | 1.1206 | 0.2992 | 0.1114 | 0.7411 |
| AC-IC | 0.029 ± 0.029 | -0.042 ± 0.022 | 0.6701 | 0.4202 | 0.6364 | 0.4320 | 0.0215 | 0.8846 |
| PBN-LC | 0.016 ± 0.131 | -0.085 ± 0.115 | 0.6673 | 0.4212 | 0.8586 | 0.3623 | 0.0466 | 0.8308 |
| Thal-Hypo | -0.040 ± 0.165 | 0.274 ± 0.205 | 0.6454 | 0.4288 | 0.1255 | 0.7259 | 2.3128 | 0.1399 |
| FOrb-Hypo | 0.197 ± 0.075 | 0.286 ± 0.128 | 0.5741 | 0.4552 | 0.1833 | 0.6720 | 0.0008 | 0.9782 |
| Thal-AC | 0.276 ± 0.073 | 0.175 ± 0.098 | 0.5448 | 0.4668 | 0.2059 | 0.6536 | 0.1837 | 0.6716 |
| Hipp-Accu | 0.372 ± 0.118 | 0.238 ± 0.132 | 0.5418 | 0.4680 | 0.1781 | 0.6764 | 0.0384 | 0.8460 |
| Amyg-LC | -0.053 ± 0.074 | -0.156 ± 0.109 | 0.4957 | 0.4874 | 0.3034 | 0.5863 | 0.8645 | 0.3607 |
| FOrb-Thal | 0.192 ± 0.074 | 0.171 ± 0.076 | 0.4601 | 0.5033 | 0.1112 | 0.7413 | 0.1456 | 0.7057 |
| int0-FOrb | 0.022 ± 0.068 | 0.065 ± 0.090 | 0.3770 | 0.5444 | 2.2465 | 0.1455 | 3.2403 | 0.0830 |
| Hypo-PBN | -0.033 ± 0.025 | -0.017 ± 0.029 | 0.3441 | 0.5623 | 0.2748 | 0.6044 | 7.1457 | 0.0126 |
| AC-FOrb | -0.253 ± 0.067 | -0.239 ± 0.124 | 0.2395 | 0.6285 | 0.1371 | 0.7140 | 7.0292 | 0.0133 |
| IC-Amyg | -0.270 ± 0.099 | -0.138 ± 0.115 | 0.2197 | 0.6430 | 0.2810 | 0.6004 | 0.4902 | 0.4898 |
| LC-Hipp | 0.131 ± 0.107 | 0.170 ± 0.096 | 0.1884 | 0.6677 | 0.6460 | 0.4286 | 0.0483 | 0.8278 |
| FOrb-AC | -0.056 ± 0.119 | -0.032 ± 0.185 | 0.1792 | 0.6754 | 0.5320 | 0.4721 | 0.3810 | 0.5422 |
| Amyg-VTA | 0.127 ± 0.109 | 0.243 ± 0.081 | 0.1078 | 0.7452 | 1.8646 | 0.1834 | 0.0127 | 0.9111 |
| PBN-Hypo | 0.102 ± 0.120 | 0.113 ± 0.148 | 0.1003 | 0.7540 | 0.1377 | 0.7134 | 0.0007 | 0.9794 |
| LC-Hypo | 0.040 ± 0.114 | 0.164 ± 0.098 | 0.0862 | 0.7713 | 2.5336 | 0.1231 | 0.0047 | 0.9460 |
| Hypo-PAG | 0.245 ± 0.073 | 0.257 ± 0.094 | 0.0668 | 0.7980 | 0.1145 | 0.7377 | 2.1968 | 0.1499 |
| Thal-IC | 0.214 ± 0.123 | 0.144 ± 0.093 | 0.0428 | 0.8377 | 0.2445 | 0.6250 | 0.6339 | 0.4329 |
| AC-Amyg | 0.071 ± 0.156 | 0.146 ± 0.137 | 0.0278 | 0.8689 | 0.0320 | 0.8593 | 0.1581 | 0.6941 |
| Hypo-Thal | 0.094 ± 0.032 | 0.113 ± 0.060 | 0.0220 | 0.8833 | 0.4593 | 0.5037 | 0.0500 | 0.8248 |
| IC-AC | 0.679 ± 0.155 | 0.732 ± 0.185 | 0.0107 | 0.9184 | 0.0979 | 0.7568 | 0.8718 | 0.3587 |
| Amyg-Hipp | 0.046 ± 0.224 | -0.089 ± 0.194 | 0.0064 | 0.9367 | 0.0435 | 0.8363 | 0.5891 | 0.4494 |
| IC-HG | 0.340 ± 0.100 | 0.318 ± 0.132 | 0.0034 | 0.9541 | 0.0004 | 0.9834 | 1.1065 | 0.3022 |
| LC-PBN | -0.059 ± 0.054 | 0.020 ± 0.073 | 0.0024 | 0.9614 | 1.0632 | 0.3116 | 5.1673 | 0.0312 |

**Table S2.6:** Variations of connectivity (DB) values in relation to group (FM vs HC) and scores on Magnification domain of PCS (2-way ANCOVA)

| **Connection** | **Group 1**  **DB values** | **Group 2**  **DB values** | **Main effect of Group** | | **Main effect of**  **PCS Magnification** | | **Interaction Effect** | |
| --- | --- | --- | --- | --- | --- | --- | --- | --- |
|  | **(mean ± s.d.)** | **(mean ± s.d.)** | **F** | **p** | **F** | **p** | **F** | **p** |
| AC-PC | 0.402 ± 0.069 | 0.679 ± 0.066 | 6.6551 | 0.0157 | 0.2667 | 0.6097 | 0.4949 | 0.4878 |
| PC-Thal | 0.025 ± 0.031 | -0.089 ± 0.035 | 6.0712 | 0.0204 | 0.0018 | 0.9660 | 5.3099 | 0.0291 |
| PAG-LC | -0.041 ± 0.092 | 0.195 ± 0.074 | 5.1115 | 0.0320 | 1.2676 | 0.2701 | 0.8656 | 0.3604 |
| Amyg-Thal | 0.339 ± 0.239 | -0.147 ± 0.203 | 3.6117 | 0.0681 | 0.6703 | 0.4201 | 5.3528 | 0.0285 |
| FOrb-LC | -0.106 ± 0.103 | 0.188 ± 0.099 | 3.4787 | 0.0731 | 0.4018 | 0.5315 | 1.3702 | 0.2520 |
| Accu-VTA | 0.036 ± 0.108 | 0.252 ± 0.054 | 3.0421 | 0.0925 | 0.2215 | 0.6417 | 0.4398 | 0.5128 |
| Amyg-IC | 0.275 ± 0.072 | 0.459 ± 0.073 | 2.5098 | 0.1248 | 0.5786 | 0.4534 | 6.5356 | 0.0165 |
| Amyg-Accu | 0.057 ± 0.033 | 0.136 ± 0.025 | 2.3614 | 0.1360 | 0.3058 | 0.5848 | 0.5658 | 0.4585 |
| Thal-HG | -0.161 ± 0.086 | -0.285 ± 0.078 | 2.3591 | 0.1362 | 2.4275 | 0.1309 | 1.1003 | 0.3035 |
| Thal-FOrb | 0.133 ± 0.085 | -0.072 ± 0.083 | 2.1891 | 0.1506 | 0.2936 | 0.5924 | 0.9327 | 0.3427 |
| LC-Thal | 0.311 ± 0.085 | 0.547 ± 0.113 | 1.9448 | 0.1745 | 0.1754 | 0.6786 | 2.2615 | 0.1442 |
| Thal-Hypo | -0.040 ± 0.165 | 0.274 ± 0.205 | 1.8220 | 0.1883 | 0.4092 | 0.5278 | 0.9247 | 0.3448 |
| IC-Amyg | -0.270 ± 0.099 | -0.138 ± 0.115 | 1.5672 | 0.2213 | 0.7190 | 0.4039 | 1.4889 | 0.2329 |
| PC-AC | 0.133 ± 0.037 | 0.285 ± 0.066 | 1.4696 | 0.2359 | 1.5158 | 0.2289 | 0.8261 | 0.3714 |
| Thal-PAG | 0.099 ± 0.120 | 0.307 ± 0.110 | 1.3719 | 0.2517 | 0.0195 | 0.8900 | 4.8675 | 0.0361 |
| FOrb-Amyg | 0.053 ± 0.077 | -0.019 ± 0.056 | 1.3112 | 0.2622 | 0.6056 | 0.4432 | 7.1717 | 0.0124 |
| VTA-Hipp | 0.098 ± 0.095 | 0.291 ± 0.108 | 1.1902 | 0.2849 | 0.3829 | 0.5412 | 0.4073 | 0.5287 |
| int2-LC | -0.014 ± 0.113 | 0.153 ± 0.075 | 1.1790 | 0.2872 | 0.1242 | 0.7273 | 0.5152 | 0.4790 |
| Amyg-Hypo | -0.021 ± 0.042 | 0.170 ± 0.109 | 1.1727 | 0.2884 | 0.3114 | 0.5814 | 2.0307 | 0.1656 |
| Thal-Hipp | 0.165 ± 0.115 | 0.296 ± 0.122 | 1.1447 | 0.2941 | 0.1611 | 0.6913 | 1.4326 | 0.2417 |
| Hypo-FOrb | -0.070 ± 0.058 | 0.087 ± 0.100 | 1.0798 | 0.3080 | 0.0009 | 0.9758 | 0.2456 | 0.6242 |
| HG-IC | 0.119 ± 0.068 | 0.048 ± 0.053 | 0.9547 | 0.3372 | 1.1261 | 0.2980 | 0.3767 | 0.5445 |
| Thal-Accu | -0.114 ± 0.075 | -0.161 ± 0.068 | 0.8810 | 0.3562 | 1.6952 | 0.2039 | 0.9514 | 0.3380 |
| Hypo-LC | -0.330 ± 0.087 | -0.214 ± 0.095 | 0.8710 | 0.3590 | 0.0133 | 0.9090 | 0.0033 | 0.9546 |
| PC-Hipp | 0.051 ± 0.037 | -0.044 ± 0.062 | 0.8453 | 0.3660 | 0.0083 | 0.9283 | 2.0176 | 0.1669 |
| Thal-AC | 0.276 ± 0.073 | 0.175 ± 0.098 | 0.7969 | 0.3799 | 0.5614 | 0.4602 | 0.6212 | 0.4375 |
| LC-Hipp | 0.131 ± 0.107 | 0.170 ± 0.096 | 0.7768 | 0.3859 | 3.0931 | 0.0900 | 0.5612 | 0.4603 |
| AC-IC | 0.029 ± 0.029 | -0.042 ± 0.022 | 0.7592 | 0.3913 | 0.7656 | 0.3893 | 0.0156 | 0.9014 |
| Thal-PC | 0.326 ± 0.094 | 0.144 ± 0.113 | 0.7318 | 0.3998 | 0.2342 | 0.6323 | 0.3087 | 0.5830 |
| FOrb-Hypo | 0.197 ± 0.075 | 0.286 ± 0.128 | 0.7307 | 0.4002 | 0.3766 | 0.5446 | 0.1550 | 0.6969 |
| PBN-Thal | 0.347 ± 0.104 | 0.074 ± 0.108 | 0.7190 | 0.4039 | 0.8386 | 0.3679 | 0.5515 | 0.4641 |
| PBN-LC | 0.016 ± 0.131 | -0.085 ± 0.115 | 0.6876 | 0.4143 | 1.0026 | 0.3256 | 1.5996 | 0.2168 |
| Amyg-Hipp | 0.046 ± 0.224 | -0.089 ± 0.194 | 0.6026 | 0.4443 | 1.2313 | 0.2769 | 0.0000 | 0.9964 |
| LC-PBN | -0.059 ± 0.054 | 0.020 ± 0.073 | 0.5506 | 0.4645 | 0.0554 | 0.8157 | 2.9687 | 0.0963 |
| PAG-PBN | 0.053 ± 0.068 | 0.077 ± 0.042 | 0.5429 | 0.4676 | 3.0691 | 0.0911 | 0.0070 | 0.9338 |
| Amyg-PAG | 0.071 ± 0.049 | 0.144 ± 0.055 | 0.4589 | 0.5039 | 0.2822 | 0.5996 | 0.9812 | 0.3307 |
| Amyg-LC | -0.053 ± 0.074 | -0.156 ± 0.109 | 0.4438 | 0.5110 | 0.2390 | 0.6288 | 1.7994 | 0.1910 |
| PBN-Hypo | 0.102 ± 0.120 | 0.113 ± 0.148 | 0.3999 | 0.5325 | 1.0237 | 0.3206 | 0.0046 | 0.9462 |
| VTA-Accu | -0.079 ± 0.138 | -0.009 ± 0.075 | 0.3980 | 0.5334 | 0.2497 | 0.6213 | 0.2657 | 0.6104 |
| FOrb-AC | -0.056 ± 0.119 | -0.032 ± 0.185 | 0.2689 | 0.6083 | 0.9665 | 0.3343 | 6.2908 | 0.0184 |
| AC-FOrb | -0.253 ± 0.067 | -0.239 ± 0.124 | 0.2333 | 0.6330 | 0.1021 | 0.7518 | 13.7740 | 0.0009 |
| Amyg-VTA | 0.127 ± 0.109 | 0.243 ± 0.081 | 0.2218 | 0.6415 | 3.4003 | 0.0762 | 0.6482 | 0.4278 |
| Hypo-PBN | -0.033 ± 0.025 | -0.017 ± 0.029 | 0.1919 | 0.6649 | 0.1063 | 0.7469 | 0.4278 | 0.5186 |
| Hipp-Amyg | -0.001 ± 0.139 | -0.135 ± 0.194 | 0.1895 | 0.6668 | 0.0418 | 0.8396 | 4.9708 | 0.0343 |
| int0-FOrb | 0.022 ± 0.068 | 0.065 ± 0.090 | 0.1622 | 0.6903 | 1.6116 | 0.2151 | 6.4819 | 0.0169 |
| Hipp-Accu | 0.372 ± 0.118 | 0.238 ± 0.132 | 0.1491 | 0.7025 | 0.0650 | 0.8007 | 0.0304 | 0.8628 |
| LC-Hypo | 0.040 ± 0.114 | 0.164 ± 0.098 | 0.1199 | 0.7318 | 3.6864 | 0.0655 | 0.0155 | 0.9018 |
| Thal-IC | 0.214 ± 0.123 | 0.144 ± 0.093 | 0.0966 | 0.7584 | 0.2140 | 0.6473 | 0.1399 | 0.7113 |
| int1-IC | -0.050 ± 0.122 | -0.149 ± 0.151 | 0.0650 | 0.8006 | 0.0173 | 0.8963 | 3.4655 | 0.0736 |
| IC-HG | 0.340 ± 0.100 | 0.318 ± 0.132 | 0.0640 | 0.8022 | 0.1610 | 0.6914 | 0.1089 | 0.7439 |
| AC-Amyg | 0.071 ± 0.156 | 0.146 ± 0.137 | 0.0505 | 0.8238 | 0.9959 | 0.3272 | 0.9772 | 0.3317 |
| FOrb-Thal | 0.192 ± 0.074 | 0.171 ± 0.076 | 0.0460 | 0.8318 | 0.3612 | 0.5529 | 1.2868 | 0.2666 |
| Hypo-PAG | 0.245 ± 0.073 | 0.257 ± 0.094 | 0.0361 | 0.8507 | 0.0567 | 0.8135 | 0.0276 | 0.8694 |
| IC-AC | 0.679 ± 0.155 | 0.732 ± 0.185 | 0.0088 | 0.9260 | 0.0012 | 0.9722 | 1.1793 | 0.2871 |
| Hypo-Thal | 0.094 ± 0.032 | 0.113 ± 0.060 | 0.0008 | 0.9776 | 0.2819 | 0.5998 | 0.5807 | 0.4526 |

**Table S2.7:** Variations of connectivity (DB) values in relation to group (FM vs HC) and scores on Rumination domain of PCS (2-way ANCOVA)

| **Connection** | **Group 1**  **DB values** | **Group 2**  **DB values** | **Main effect of Group** | | **Main effect of**  **PCS Rumination** | | **Interaction Effect** | |
| --- | --- | --- | --- | --- | --- | --- | --- | --- |
|  | **(mean ± s.d.)** | **(mean ± s.d.)** | **F** | **p** | **F** | **p** | **F** | **p** |
| AC-PC | 0.402 ± 0.069 | 0.679 ± 0.066 | 6.5025 | 0.0168 | 0.1512 | 0.7005 | 0.8266 | 0.3713 |
| PC-Thal | 0.025 ± 0.031 | -0.089 ± 0.035 | 5.8713 | 0.0224 | 0.0011 | 0.9738 | 2.3804 | 0.1345 |
| PAG-LC | -0.041 ± 0.092 | 0.195 ± 0.074 | 5.0185 | 0.0335 | 1.1880 | 0.2854 | 0.8328 | 0.3695 |
| Amyg-Accu | 0.057 ± 0.033 | 0.136 ± 0.025 | 4.6413 | 0.0403 | 2.9130 | 0.0993 | 0.0032 | 0.9552 |
| Thal-PAG | 0.099 ± 0.120 | 0.307 ± 0.110 | 4.5114 | 0.0430 | 3.2547 | 0.0824 | 0.5466 | 0.4661 |
| Amyg-Thal | 0.339 ± 0.239 | -0.147 ± 0.203 | 3.0829 | 0.0905 | 0.4150 | 0.5249 | 2.8889 | 0.1007 |
| FOrb-LC | -0.106 ± 0.103 | 0.188 ± 0.099 | 3.0109 | 0.0941 | 0.0727 | 0.7895 | 3.1645 | 0.0865 |
| PC-AC | 0.133 ± 0.037 | 0.285 ± 0.066 | 2.8971 | 0.1002 | 0.1278 | 0.7235 | 2.3945 | 0.1334 |
| Thal-HG | -0.161 ± 0.086 | -0.285 ± 0.078 | 2.4775 | 0.1271 | 2.8789 | 0.1012 | 0.1746 | 0.6794 |
| Thal-FOrb | 0.133 ± 0.085 | -0.072 ± 0.083 | 2.3267 | 0.1388 | 0.2176 | 0.6446 | 0.0585 | 0.8107 |
| Accu-VTA | 0.036 ± 0.108 | 0.252 ± 0.054 | 2.1320 | 0.1558 | 0.0107 | 0.9184 | 0.1045 | 0.7490 |
| VTA-Hipp | 0.098 ± 0.095 | 0.291 ± 0.108 | 1.8076 | 0.1900 | 1.2549 | 0.2725 | 0.2491 | 0.6218 |
| Thal-Hypo | -0.040 ± 0.165 | 0.274 ± 0.205 | 1.7485 | 0.1972 | 0.3712 | 0.5474 | 0.2162 | 0.6457 |
| Thal-PC | 0.326 ± 0.094 | 0.144 ± 0.113 | 1.5885 | 0.2183 | 0.0056 | 0.9407 | 9.9121 | 0.0040 |
| Amyg-PAG | 0.071 ± 0.049 | 0.144 ± 0.055 | 1.5252 | 0.2275 | 0.3414 | 0.5639 | 0.0203 | 0.8878 |
| Amyg-IC | 0.275 ± 0.072 | 0.459 ± 0.073 | 1.3410 | 0.2570 | 0.0114 | 0.9156 | 1.3491 | 0.2556 |
| AC-IC | 0.029 ± 0.029 | -0.042 ± 0.022 | 1.2839 | 0.2671 | 0.1550 | 0.6969 | 0.0490 | 0.8265 |
| Hipp-Accu | 0.372 ± 0.118 | 0.238 ± 0.132 | 1.2608 | 0.2714 | 1.4002 | 0.2470 | 0.6376 | 0.4316 |
| Hypo-LC | -0.330 ± 0.087 | -0.214 ± 0.095 | 1.2463 | 0.2741 | 0.0429 | 0.8375 | 0.3705 | 0.5478 |
| Amyg-Hypo | -0.021 ± 0.042 | 0.170 ± 0.109 | 1.2455 | 0.2743 | 0.2247 | 0.6393 | 0.3856 | 0.5398 |
| LC-Thal | 0.311 ± 0.085 | 0.547 ± 0.113 | 1.1078 | 0.3019 | 0.0385 | 0.8460 | 0.0266 | 0.8715 |
| Thal-Accu | -0.114 ± 0.075 | -0.161 ± 0.068 | 0.9236 | 0.3451 | 1.9730 | 0.1715 | 0.1293 | 0.7220 |
| Hypo-FOrb | -0.070 ± 0.058 | 0.087 ± 0.100 | 0.8877 | 0.3544 | 0.0361 | 0.8508 | 0.0089 | 0.9254 |
| int1-IC | -0.050 ± 0.122 | -0.149 ± 0.151 | 0.8737 | 0.3582 | 2.3232 | 0.1391 | 0.3751 | 0.5453 |
| IC-Amyg | -0.270 ± 0.099 | -0.138 ± 0.115 | 0.7492 | 0.3943 | 0.0180 | 0.8942 | 0.1191 | 0.7327 |
| PC-Hipp | 0.051 ± 0.037 | -0.044 ± 0.062 | 0.6660 | 0.4216 | 0.0998 | 0.7545 | 1.3468 | 0.2560 |
| PBN-Thal | 0.347 ± 0.104 | 0.074 ± 0.108 | 0.5857 | 0.4507 | 1.3606 | 0.2536 | 0.0024 | 0.9616 |
| VTA-Accu | -0.079 ± 0.138 | -0.009 ± 0.075 | 0.5744 | 0.4551 | 0.5841 | 0.4513 | 0.3539 | 0.5569 |
| HG-IC | 0.119 ± 0.068 | 0.048 ± 0.053 | 0.4115 | 0.5266 | 0.1526 | 0.6992 | 2.7116 | 0.1112 |
| int2-LC | -0.014 ± 0.113 | 0.153 ± 0.075 | 0.3785 | 0.5436 | 0.3849 | 0.5402 | 0.2604 | 0.6140 |
| PBN-LC | 0.016 ± 0.131 | -0.085 ± 0.115 | 0.3602 | 0.5534 | 0.3544 | 0.5566 | 0.0259 | 0.8733 |
| Thal-Hipp | 0.165 ± 0.115 | 0.296 ± 0.122 | 0.3587 | 0.5542 | 0.4833 | 0.4929 | 5.3050 | 0.0292 |
| FOrb-Hypo | 0.197 ± 0.075 | 0.286 ± 0.128 | 0.3411 | 0.5641 | 0.0037 | 0.9520 | 1.1065 | 0.3022 |
| Thal-AC | 0.276 ± 0.073 | 0.175 ± 0.098 | 0.3086 | 0.5831 | 0.0095 | 0.9231 | 0.2427 | 0.6262 |
| Hipp-Amyg | -0.001 ± 0.139 | -0.135 ± 0.194 | 0.3064 | 0.5845 | 0.2393 | 0.6287 | 0.7558 | 0.3923 |
| FOrb-AC | -0.056 ± 0.119 | -0.032 ± 0.185 | 0.2975 | 0.5899 | 1.1744 | 0.2881 | 4.1989 | 0.0503 |
| PBN-Hypo | 0.102 ± 0.120 | 0.113 ± 0.148 | 0.2297 | 0.6356 | 0.5543 | 0.4630 | 0.0083 | 0.9282 |
| AC-FOrb | -0.253 ± 0.067 | -0.239 ± 0.124 | 0.2283 | 0.6366 | 0.1605 | 0.6918 | 3.6757 | 0.0658 |
| FOrb-Thal | 0.192 ± 0.074 | 0.171 ± 0.076 | 0.1939 | 0.6632 | 0.0234 | 0.8795 | 0.0120 | 0.9138 |
| FOrb-Amyg | 0.053 ± 0.077 | -0.019 ± 0.056 | 0.1801 | 0.6747 | 0.3705 | 0.5478 | 4.2671 | 0.0486 |
| Amyg-LC | -0.053 ± 0.074 | -0.156 ± 0.109 | 0.1685 | 0.6847 | 0.0000 | 0.9945 | 0.9510 | 0.3381 |
| AC-Amyg | 0.071 ± 0.156 | 0.146 ± 0.137 | 0.1587 | 0.6935 | 0.0511 | 0.8229 | 1.2326 | 0.2767 |
| int0-FOrb | 0.022 ± 0.068 | 0.065 ± 0.090 | 0.1273 | 0.7240 | 1.4283 | 0.2424 | 0.5099 | 0.4813 |
| LC-Hipp | 0.131 ± 0.107 | 0.170 ± 0.096 | 0.1179 | 0.7339 | 0.5493 | 0.4650 | 0.9872 | 0.3292 |
| Hypo-PAG | 0.245 ± 0.073 | 0.257 ± 0.094 | 0.1179 | 0.7340 | 0.3067 | 0.5843 | 2.2811 | 0.1426 |
| IC-HG | 0.340 ± 0.100 | 0.318 ± 0.132 | 0.0959 | 0.7592 | 0.2824 | 0.5995 | 1.3373 | 0.2576 |
| LC-PBN | -0.059 ± 0.054 | 0.020 ± 0.073 | 0.0799 | 0.7795 | 0.4797 | 0.4945 | 1.7130 | 0.2016 |
| PAG-PBN | 0.053 ± 0.068 | 0.077 ± 0.042 | 0.0774 | 0.7829 | 0.7911 | 0.3816 | 0.0811 | 0.7780 |
| Hypo-PBN | -0.033 ± 0.025 | -0.017 ± 0.029 | 0.0442 | 0.8351 | 0.0207 | 0.8866 | 1.6705 | 0.2071 |
| Thal-IC | 0.214 ± 0.123 | 0.144 ± 0.093 | 0.0302 | 0.8634 | 0.2576 | 0.6159 | 0.2656 | 0.6105 |
| Hypo-Thal | 0.094 ± 0.032 | 0.113 ± 0.060 | 0.0245 | 0.8767 | 0.0322 | 0.8589 | 0.1657 | 0.6872 |
| IC-AC | 0.679 ± 0.155 | 0.732 ± 0.185 | 0.0158 | 0.9009 | 0.0105 | 0.9192 | 0.2338 | 0.6326 |
| Amyg-VTA | 0.127 ± 0.109 | 0.243 ± 0.081 | 0.0119 | 0.9138 | 1.3535 | 0.2549 | 0.0754 | 0.7857 |
| Amyg-Hipp | 0.046 ± 0.224 | -0.089 ± 0.194 | 0.0040 | 0.9498 | 0.0852 | 0.7727 | 0.0272 | 0.8703 |
| LC-Hypo | 0.040 ± 0.114 | 0.164 ± 0.098 | 0.0012 | 0.9723 | 1.8381 | 0.1864 | 0.1432 | 0.7081 |

**Table S2.8:** Variations of connectivity (DB) values in relation to group (FM vs HC) and scores on COMPASS-31 scores (2-way ANCOVA)

| **Connection** | **Group 1**  **DB values** | **Group 2**  **DB values** | **Main effect of Group** | | **Main effect of**  **COMPASS-31 scores** | | **Interaction Effect** | |
| --- | --- | --- | --- | --- | --- | --- | --- | --- |
|  | **(mean ± s.d.)** | **(mean ± s.d.)** | **F** | **p** | **F** | **p** | **F** | **p** |
| AC-IC | 0.029 ± 0.029 | -0.042 ± 0.022 | 11.2209 | 0.0022 | 7.4962 | 0.0103 | 0.1057 | 0.7474 |
| IC-HG | 0.340 ± 0.100 | 0.318 ± 0.132 | 7.0710 | 0.0124 | 12.0972 | 0.0016 | 2.0496 | 0.1626 |
| Amyg-Hypo | -0.021 ± 0.042 | 0.170 ± 0.109 | 5.0135 | 0.0327 | 1.8142 | 0.1881 | 4.5763 | 0.0407 |
| Amyg-IC | 0.275 ± 0.072 | 0.459 ± 0.073 | 4.0155 | 0.0542 | 1.2738 | 0.2680 | 0.9298 | 0.3426 |
| Thal-FOrb | 0.133 ± 0.085 | -0.072 ± 0.083 | 3.1624 | 0.0855 | 0.8561 | 0.3622 | 0.0351 | 0.8526 |
| AC-PC | 0.402 ± 0.069 | 0.679 ± 0.066 | 3.0007 | 0.0935 | 0.0304 | 0.8627 | 1.3583 | 0.2530 |
| PBN-Hypo | 0.102 ± 0.120 | 0.113 ± 0.148 | 2.6436 | 0.1144 | 4.6698 | 0.0388 | 0.4730 | 0.4969 |
| Thal-Hypo | -0.040 ± 0.165 | 0.274 ± 0.205 | 2.4049 | 0.1314 | 1.0041 | 0.3243 | 2.5546 | 0.1205 |
| PC-Thal | 0.025 ± 0.031 | -0.089 ± 0.035 | 2.1105 | 0.1567 | 0.0203 | 0.8875 | 0.1436 | 0.7074 |
| Hypo-LC | -0.330 ± 0.087 | -0.214 ± 0.095 | 1.8074 | 0.1889 | 1.0641 | 0.3105 | 0.2261 | 0.6378 |
| Thal-AC | 0.276 ± 0.073 | 0.175 ± 0.098 | 1.7301 | 0.1984 | 0.9558 | 0.3361 | 5.7253 | 0.0232 |
| LC-Hypo | 0.040 ± 0.114 | 0.164 ± 0.098 | 1.7203 | 0.1996 | 1.0958 | 0.3036 | 2.6455 | 0.1143 |
| IC-Amyg | -0.270 ± 0.099 | -0.138 ± 0.115 | 1.6571 | 0.2078 | 0.9508 | 0.3373 | 0.7872 | 0.3820 |
| Thal-Accu | -0.114 ± 0.075 | -0.161 ± 0.068 | 1.5904 | 0.2170 | 4.5737 | 0.0407 | 0.5543 | 0.4624 |
| Thal-PAG | 0.099 ± 0.120 | 0.307 ± 0.110 | 1.5486 | 0.2230 | 0.3647 | 0.5504 | 0.0116 | 0.9149 |
| FOrb-Amyg | 0.053 ± 0.077 | -0.019 ± 0.056 | 1.4476 | 0.2383 | 0.9838 | 0.3292 | 0.0717 | 0.7907 |
| Accu-VTA | 0.036 ± 0.108 | 0.252 ± 0.054 | 1.3287 | 0.2581 | 0.0176 | 0.8954 | 0.1677 | 0.6850 |
| PC-AC | 0.133 ± 0.037 | 0.285 ± 0.066 | 1.3243 | 0.2589 | 0.0831 | 0.7751 | 0.1653 | 0.6873 |
| PAG-LC | -0.041 ± 0.092 | 0.195 ± 0.074 | 1.1883 | 0.2844 | 0.0781 | 0.7818 | 3.4595 | 0.0727 |
| LC-PBN | -0.059 ± 0.054 | 0.020 ± 0.073 | 1.1582 | 0.2904 | 0.4522 | 0.5064 | 1.2905 | 0.2649 |
| Amyg-Thal | 0.339 ± 0.239 | -0.147 ± 0.203 | 1.0669 | 0.3099 | 0.0064 | 0.9370 | 1.0441 | 0.3150 |
| FOrb-Hypo | 0.197 ± 0.075 | 0.286 ± 0.128 | 1.0158 | 0.3216 | 3.8567 | 0.0589 | 1.2436 | 0.2736 |
| Amyg-PAG | 0.071 ± 0.049 | 0.144 ± 0.055 | 1.0129 | 0.3223 | 5.3860 | 0.0273 | 1.3044 | 0.2624 |
| Hypo-FOrb | -0.070 ± 0.058 | 0.087 ± 0.100 | 0.9375 | 0.3407 | 0.0016 | 0.9686 | 2.2785 | 0.1416 |
| PC-Hipp | 0.051 ± 0.037 | -0.044 ± 0.062 | 0.9178 | 0.3457 | 0.0096 | 0.9224 | 0.0639 | 0.8022 |
| int2-LC | -0.014 ± 0.113 | 0.153 ± 0.075 | 0.8846 | 0.3544 | 5.6852 | 0.0236 | 0.0035 | 0.9532 |
| AC-FOrb | -0.253 ± 0.067 | -0.239 ± 0.124 | 0.8814 | 0.3553 | 1.3992 | 0.2461 | 0.8114 | 0.3749 |
| FOrb-AC | -0.056 ± 0.119 | -0.032 ± 0.185 | 0.7899 | 0.3812 | 1.2368 | 0.2749 | 1.2346 | 0.2753 |
| int1-IC | -0.050 ± 0.122 | -0.149 ± 0.151 | 0.6143 | 0.4393 | 2.3756 | 0.1337 | 0.8389 | 0.3670 |
| FOrb-LC | -0.106 ± 0.103 | 0.188 ± 0.099 | 0.6092 | 0.4412 | 0.5451 | 0.4661 | 1.1810 | 0.2858 |
| Amyg-VTA | 0.127 ± 0.109 | 0.243 ± 0.081 | 0.4556 | 0.5049 | 0.0397 | 0.8434 | 0.9608 | 0.3348 |
| AC-Amyg | 0.071 ± 0.156 | 0.146 ± 0.137 | 0.4539 | 0.5056 | 1.5735 | 0.2194 | 4.8901 | 0.0348 |
| LC-Thal | 0.311 ± 0.085 | 0.547 ± 0.113 | 0.4517 | 0.5067 | 0.3468 | 0.5603 | 0.7883 | 0.3817 |
| Thal-HG | -0.161 ± 0.086 | -0.285 ± 0.078 | 0.4344 | 0.5149 | 3.5172 | 0.0705 | 1.3572 | 0.2532 |
| IC-AC | 0.679 ± 0.155 | 0.732 ± 0.185 | 0.4253 | 0.5192 | 0.4708 | 0.4979 | 2.9867 | 0.0942 |
| Amyg-Accu | 0.057 ± 0.033 | 0.136 ± 0.025 | 0.4192 | 0.5223 | 0.5877 | 0.4493 | 1.0971 | 0.3033 |
| Hipp-Amyg | -0.001 ± 0.139 | -0.135 ± 0.194 | 0.3589 | 0.5536 | 0.0994 | 0.7547 | 0.0130 | 0.9101 |
| LC-Hipp | 0.131 ± 0.107 | 0.170 ± 0.096 | 0.3224 | 0.5744 | 1.0164 | 0.3214 | 0.0349 | 0.8530 |
| PBN-Thal | 0.347 ± 0.104 | 0.074 ± 0.108 | 0.2808 | 0.6001 | 0.7427 | 0.3956 | 0.0066 | 0.9356 |
| Hypo-PAG | 0.245 ± 0.073 | 0.257 ± 0.094 | 0.2792 | 0.6011 | 0.6812 | 0.4157 | 8.6215 | 0.0063 |
| PBN-LC | 0.016 ± 0.131 | -0.085 ± 0.115 | 0.2149 | 0.6463 | 0.0180 | 0.8942 | 0.8646 | 0.3599 |
| HG-IC | 0.119 ± 0.068 | 0.048 ± 0.053 | 0.1598 | 0.6921 | 0.0229 | 0.8808 | 0.9448 | 0.3388 |
| Amyg-LC | -0.053 ± 0.074 | -0.156 ± 0.109 | 0.1590 | 0.6929 | 0.0254 | 0.8745 | 0.0310 | 0.8614 |
| Amyg-Hipp | 0.046 ± 0.224 | -0.089 ± 0.194 | 0.1526 | 0.6988 | 0.0191 | 0.8909 | 1.3130 | 0.2609 |
| Hypo-PBN | -0.033 ± 0.025 | -0.017 ± 0.029 | 0.1414 | 0.7095 | 0.0232 | 0.8799 | 2.0894 | 0.1587 |
| VTA-Hipp | 0.098 ± 0.095 | 0.291 ± 0.108 | 0.1205 | 0.7309 | 2.9851 | 0.0943 | 2.0549 | 0.1621 |
| Thal-Hipp | 0.165 ± 0.115 | 0.296 ± 0.122 | 0.0882 | 0.7685 | 1.2034 | 0.2814 | 0.6040 | 0.4432 |
| int0-FOrb | 0.022 ± 0.068 | 0.065 ± 0.090 | 0.0646 | 0.8012 | 0.4816 | 0.4930 | 0.6192 | 0.4375 |
| Thal-IC | 0.214 ± 0.123 | 0.144 ± 0.093 | 0.0572 | 0.8127 | 0.0045 | 0.9472 | 2.3113 | 0.1389 |
| Hipp-Accu | 0.372 ± 0.118 | 0.238 ± 0.132 | 0.0507 | 0.8234 | 0.9404 | 0.3399 | 0.0248 | 0.8759 |
| VTA-Accu | -0.079 ± 0.138 | -0.009 ± 0.075 | 0.0256 | 0.8740 | 0.0221 | 0.8828 | 0.6888 | 0.4131 |
| PAG-PBN | 0.053 ± 0.068 | 0.077 ± 0.042 | 0.0143 | 0.9056 | 0.1724 | 0.6810 | 0.9683 | 0.3330 |
| FOrb-Thal | 0.192 ± 0.074 | 0.171 ± 0.076 | 0.0097 | 0.9221 | 0.0934 | 0.7620 | 0.2798 | 0.6007 |
| Thal-PC | 0.326 ± 0.094 | 0.144 ± 0.113 | 0.0030 | 0.9568 | 1.4608 | 0.2362 | 1.4643 | 0.2357 |
| Hypo-Thal | 0.094 ± 0.032 | 0.113 ± 0.060 | 0.0009 | 0.9761 | 0.1015 | 0.7522 | 3.3835 | 0.0758 |

**Table S2.9:** The magnitude of the intial rise in BOLD signal in relation to group (FM vs HC) and pain ratings (2-way ANCOVA)

| **Region** | **Main effect of Group** | | **Main effect of**  **pain ratings** | | **Interaction Effect** | |
| --- | --- | --- | --- | --- | --- | --- |
|  | **F** | **p** | **F** | **F** | **p** | **F** |
| AC | 14.1310 | 0.0007 | 0.6116 | 0.4403 | 2.6003 | 0.1173 |
| PC | 10.0285 | 0.0035 | 1.5544 | 0.2221 | 0.0135 | 0.9081 |
| FOrb | 8.4514 | 0.0068 | 0.0215 | 0.8843 | 0.8330 | 0.3687 |
| Thalamus | 5.2209 | 0.0296 | 0.2072 | 0.6522 | 0.1047 | 0.7485 |
| HG | 5.1954 | 0.0299 | 0.1194 | 0.7321 | 7.7638 | 0.0092 |
| IC | 5.0087 | 0.0328 | 0.0228 | 0.8811 | 0.2866 | 0.5964 |
| Accumbens | 3.5908 | 0.0678 | 1.0153 | 0.3217 | 0.1158 | 0.7360 |
| Amygdala | 3.1761 | 0.0849 | 0.1861 | 0.6693 | 0.3992 | 0.5323 |
| Hippocampus | 2.9538 | 0.0960 | 0.3632 | 0.5513 | 0.3984 | 0.5327 |
| Hypothalamus | 2.3724 | 0.1340 | 0.1823 | 0.6725 | 0.0713 | 0.7912 |
| PAG | 1.6084 | 0.2145 | 0.6978 | 0.4101 | 2.1616 | 0.1519 |
| VTA | 1.6033 | 0.2152 | 0.4514 | 0.5068 | 5.2087 | 0.0297 |
| LC | 0.4279 | 0.5180 | 0.3145 | 0.5791 | 0.1874 | 0.6682 |
| PBN | 0.0108 | 0.9179 | 3.0998 | 0.0885 | 1.5858 | 0.2176 |

**Table S2.10:** The magnitude of the intial rise in BOLD signal in relation to group (FM vs HC) and STAI-Y-1 score (2-way ANCOVA)

| **Region** | **Main effect of Group** | | **Main effect of**  **STAI-Y-1 score** | | **Interaction Effect** | |
| --- | --- | --- | --- | --- | --- | --- |
|  | **F** | **p** | **F** | **p** | **F** | **p** |
| AC | 13.8879 | 0.0008 | 2.9042 | 0.0987 | 0.1517 | 0.6997 |
| PC | 10.8407 | 0.0025 | 8.8166 | 0.0058 | 0.6805 | 0.4159 |
| FOrb | 10.1972 | 0.0033 | 3.3803 | 0.0759 | 0.2309 | 0.6343 |
| Thalamus | 8.2336 | 0.0075 | 3.0652 | 0.0902 | 0.1805 | 0.6740 |
| HG | 7.0671 | 0.0125 | 4.1785 | 0.0498 | 0.0551 | 0.8160 |
| IC | 6.2557 | 0.0181 | 7.2587 | 0.0114 | 0.1036 | 0.7498 |
| Accumbens | 5.5941 | 0.0247 | 5.0506 | 0.0321 | 0.4089 | 0.5274 |
| Amygdala | 4.7054 | 0.0381 | 7.3192 | 0.0111 | 0.2431 | 0.6255 |
| Hippocampus | 3.5567 | 0.0690 | 1.3071 | 0.2620 | 0.3793 | 0.5426 |
| Hypothalamus | 3.0155 | 0.0927 | 4.6179 | 0.0398 | 0.9135 | 0.3468 |
| PAG | 2.3551 | 0.1354 | 8.8995 | 0.0056 | 0.0624 | 0.8045 |
| VTA | 2.3342 | 0.1370 | 0.0025 | 0.9607 | 2.0106 | 0.1665 |
| LC | 0.8434 | 0.3658 | 10.6638 | 0.0027 | 0.1699 | 0.6832 |
| PBN | 0.2530 | 0.6187 | 3.3648 | 0.0765 | 1.4047 | 0.2452 |

**Table S2.11:** The magnitude of the intial rise in BOLD signal in relation to group (FM vs HC) and STAI-Y-2 score (2-way ANCOVA)

| **Region** | **Main effect of Group** | | **Main effect of**  **STAI-Y-2 score** | | **Interaction Effect** | |
| --- | --- | --- | --- | --- | --- | --- |
|  | **F** | **p** | **F** | **p** | **F** | **p** |
| AC | 10.5916 | 0.0029 | 2.2232 | 0.1468 | 2.9251 | 0.0979 |
| PC | 9.3004 | 0.0049 | 3.0290 | 0.0924 | 0.3572 | 0.5547 |
| FOrb | 8.9378 | 0.0056 | 0.2585 | 0.6150 | 1.2878 | 0.2657 |
| Thalamus | 8.5809 | 0.0066 | 6.7482 | 0.0146 | 2.5141 | 0.1237 |
| HG | 5.4303 | 0.0270 | 5.1331 | 0.0311 | 0.6153 | 0.4391 |
| IC | 5.3753 | 0.0277 | 10.8800 | 0.0026 | 1.1859 | 0.2851 |
| Accumbens | 4.4651 | 0.0433 | 6.6969 | 0.0149 | 2.6268 | 0.1159 |
| Amygdala | 4.0540 | 0.0534 | 2.1123 | 0.1569 | 2.5079 | 0.1241 |
| Hippocampus | 2.8903 | 0.0998 | 2.1124 | 0.1568 | 1.7696 | 0.1938 |
| Hypothalamus | 2.8757 | 0.1006 | 0.3727 | 0.5463 | 0.1154 | 0.7366 |
| PAG | 1.8935 | 0.1793 | 7.0721 | 0.0126 | 4.7037 | 0.0384 |
| VTA | 1.2505 | 0.2726 | 6.5891 | 0.0157 | 0.9727 | 0.3322 |
| LC | 1.0512 | 0.3137 | 0.3899 | 0.5372 | 0.0993 | 0.7550 |
| PBN | 0.0592 | 0.8095 | 7.3803 | 0.0110 | 0.0207 | 0.8866 |

**Table S2.12:** The magnitude of the intial rise in BOLD signal in relation to group (FM vs HC) and PCS Total scores (2-way ANCOVA)

| **Region** | **Main effect of Group** | | **Main effect of**  **PCS Total score** | | **Interaction Effect** | |
| --- | --- | --- | --- | --- | --- | --- |
|  | **F** | **p** | **F** | **p** | **F** | **p** |
| AC | 9.8922 | 0.0037 | 1.3079 | 0.2618 | 0.1177 | 0.7339 |
| PC | 8.0315 | 0.0081 | 0.6111 | 0.4405 | 0.4834 | 0.4922 |
| FOrb | 7.6460 | 0.0096 | 0.5005 | 0.4848 | 0.4239 | 0.5199 |
| Thalamus | 6.5772 | 0.0156 | 0.0029 | 0.9576 | 2.4857 | 0.1254 |
| HG | 6.5676 | 0.0156 | 1.0624 | 0.3109 | 0.8390 | 0.3670 |
| IC | 5.5524 | 0.0252 | 0.2259 | 0.6380 | 1.0872 | 0.3054 |
| Accumbens | 5.5467 | 0.0253 | 2.8237 | 0.1033 | 1.6949 | 0.2029 |
| Amygdala | 4.7070 | 0.0381 | 0.1372 | 0.7137 | 0.8958 | 0.3515 |
| Hippocampus | 4.5458 | 0.0413 | 1.2777 | 0.2673 | 0.0745 | 0.7868 |
| Hypothalamus | 2.4476 | 0.1282 | 3.8327 | 0.0596 | 1.8346 | 0.1857 |
| PAG | 1.6781 | 0.2051 | 0.3548 | 0.5559 | 0.6825 | 0.4153 |
| VTA | 1.2862 | 0.2657 | 0.8191 | 0.3727 | 0.1584 | 0.6935 |
| LC | 1.1794 | 0.2861 | 2.4035 | 0.1315 | 0.8769 | 0.3565 |
| PBN | 0.9332 | 0.3418 | 0.9007 | 0.3502 | 0.0276 | 0.8691 |
