# Supplementary material for: Evidence of a persistent altered neural state in people with fibromyalgia syndrome during functional MRI studies and its relationship with pain and anxiety
Source: PLoS One. 2025 Jan 24;20(1):e0316672. doi: 10.1371/journal.pone.0316672 (PMC11759356; doi:10.1371/journal.pone.0316672)
Supplement: S3 Appendix — (DOCX) [file pone.0316672.s003.docx]

**S3 Appendix: *Comparisons of BOLD responses for participants stratified according to pain sensitivity***

Average time-course responses are shown in Figure S2.1 for sub-groups of participants with fibromyalgia (FM) stratified into high, medium, and low pain sensitivity for several regions. These plots demonstrate the consistency of the initial rise, and that it does not appear to have any consistent dependence on pain sensitivity. However, at times when participants were informed of the stimulus type, and after the onsets and offsets of stimulation, these plots demonstrate patterns of variation that appear to depend on pain sensitivity. Particularly in the insular cortex, thalamus, and amygdala, the increased BOLD signal at the onset of stimulation varies in order of pain sensitivity. Notably, the increase in BOLD signal after the onset of stimulation is highest for the lowest sensitivity group. It is also notable that the larger initial rise in BOLD signal in the FM group corresponds with larger BOLD signal changes at the onset of the stimulation in the FM group, yet in the FM group pain sensitivity is higher.


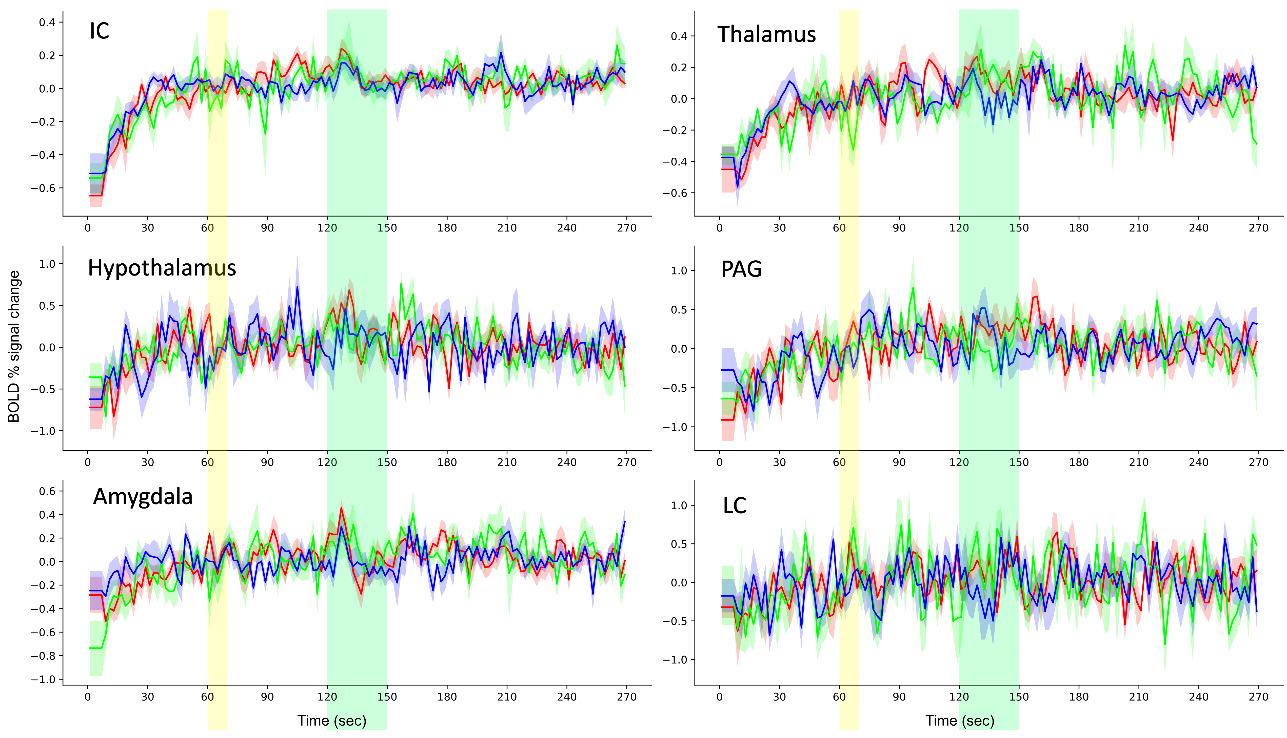


**Figure S3.1:** Plots of BOLD time-course responses in selected regions for participants with fibromyalgia during the noxious stimulation condition. Time-courses were grouped by participants according to pain sensitivity and were averaged. Red lines correspond with the lowest pain sensitivity (0.675 ± 0.111), green with moderate sensitivity (0.913 ± 0.045), and blue with highest sensitivity (1.188 ± 0.126). (pain sensitivity = pain rating / temperature). The period when participants were informed of the stimulus type is indicated with a yellow band, and the stimulation period is indicated with a green band.
