# Supplementary material for: Evidence of a persistent altered neural state in people with fibromyalgia syndrome during functional MRI studies and its relationship with pain and anxiety
Source: PLoS One. 2025 Jan 24;20(1):e0316672. doi: 10.1371/journal.pone.0316672 (PMC11759356; doi:10.1371/journal.pone.0316672)
Supplement: Attachment — Submitted filename: Responses_to_Reviews from PLOS One Sept2024.docx [file pone.0316672.s004.docx]

**Responses to Reviews from PLOS One (PONE-D-24-17827R1)**

We appreciate the time taken by the reviewers. We found the comments and suggestions to be very helpful and we believe that the revisions have improved the quality and clarity of the manuscript. We have attempted to fully address each of the reviewers’ points, as detailed below. However, most of the points raised suggested that we add information or details, resulting in a substantial increase in the length of the manuscript. We have attempted to provide the suggested information but in places we felt that we had to balance the responses with keeping the manuscript focussed on the topic and as concise as possible. We have also moved some information to supplementary material as suggested, and to reduce the length of the primary text.

Please find the detailed responses to each point below, following the reviewers’ comments (in italics) which we have included to be clear which points were are responding to for each revision.

***Reviewer #1****: Stroman et al. examined task-fMRI data from a painful stimulation paradigm in women with fibromyalgia and healthy females using a novel approach developed by the authors. The way I understood it, this approach tests connectivity weights of input and output signals in an anatomically informed network model. They found differences between groups and associations with questionnaires. The main finding was a signal increase at the beginning of each scan that was more pronounced in women with fibromyalgia. This signal increase was associated with anxiety. In a similar sample the authors also found larger pupil sizes in women with fibromyalgia. They conclude that people with fibromyalgia may have an exaggerated stress response.*

*More research about the pathophysiology of fibromyalgia is needed. The present study is interesting, and the reasons to not make the underlying data publicly available are sound. However, I have several comments and questions:*

*Although previous work is referenced for characterization of the study sample, a summary of key demographic data should be presented in this manuscript as well.*

In the “Participants” subsection of the Methods we have included information about the participants being all female, and the range and average ages for the HC and FM groups. We have now added more information about the pain ratings and temperatures of the stimuli as well, as follows:

“As described previously (1), the calibrated temperature was significantly different (p = 2.55 x 10^-5^) between the two groups and averaged 50.89 ± 1.04 °C for the HC group and 47.51 ± 2.72 °C for the FM group. The average pain ratings to the last contact in each set of 10 contacts were 38.93 ± 12.07 and 44.22 ± 12.04 on a 0-100 scale, for the HC and FM groups respectively, and were not signficantly different. The temperatures and pain ratings demonstrate the higher heat pain sensitivity in the FM group.”

*The authors report that scans took place in two different (albeit very similar) scanners or scanner versions. Were the different scanners accounted for in the analyses? What proportion of the data was acquired in each configuration?*

We have added the following sentences to the Methods section to provide this information:

“Approximately midway through data collection, the MRI system was upgraded from a Siemens Trio to a Siemens Prisma. Test scans involving our experimental paradigm were performed on two participants before and after the upgrade, and the data quality were compared. No significant differences in BOLD activity between the datasets were found.”

*For normalization, briefly describe the normalization approach. Reference the original articles for the applied approach, not your own review discussing different approaches. Which atlas(es) were used to select the anatomical regions?*

We did not reference a review article. We referenced our article that provided a detailed description and validation of our methods (Stroman PW, Powers JM, Ioachim G. Proof-of-concept of a novel structural equation modelling approach for the analysis of functional magnetic resonance imaging data applied to investigate individual differences in human pain responses. Hum Brain Mapp. 2023;44(6):2523-585 42.). It is necessary to rely on cited work that provide essential background in order to keep a paper focussed on the current topic. Nonetheless, in order to respond to the reviewer’s comment, we have added the following information to the Methods section:

“The reference images were created by combining the MNI152 template from the Statistical Parametric Mapping (SPM12) software package (38) and the PAM50 template, as described by De Leener et al. (39). Anatomical region-of-interest maps were defined from multiple sources, including anatomical descriptions, freely shared anatomical maps, and the CONN15e software package (40-50). These sources were combined to create a single anatomical map. The normalization procedure employed different methods for the brain, and for brainstem and cord regions. For brain regions, the normalization process uses the python package “dipy” (https://dipy.org/documentation/1.5.0/documentation/ ) which is based on the ANTs (Advanced Normalization Tools) software (51, 52). Normalization of brainstem and spinal cord regions has been described previously (26, 53) and involves mapping sections of the template to the image data for regions with distinct anatomical features. These region positions then guide the identification of successive sections of the spinal cord based on cross-correlation, progressing away from the brainstem, in a manner that maintains the distance along the cord. The normalization procedure is based on the premise that the cord anatomy is likely much more consistent in size across people, than is the spine anatomy (44). The normalization was then fine-tuned using the Medical Image Registration (MIRT) toolbox, translated into python (54).”

*It is not clear to me whether just one subregion per region was used in all analyses. The subregions that were used for SAPM should be identified for each region (e.g. in a supplement).*

We state in the Methods section (lines 321 to 322) that:

“In order to compare results from the brain, and from brainstem/cord, the same sub-regions were used for SAPM analyses, for the regions that are common to both network models.”

To be more clear we have expanded this to read:

“This combination of sub-regions therefore has BOLD time-series responses that fit the network model better than other combinations of sub-regions. This same set of sub-regions was then used for all subsequent analyses for consistency. In order to compare results from the brain, and from brainstem/cord, the same sub-regions were used for SAPM analyses for the regions that are common to both network models.”

Showing the 3D anatomical subregions for all 14 regions that were included in the brain network, and the additional 6 regions in the brainstem/cord network that were not included in the brain would mean that we need to display 20 regions. Multiple images are needed to depict the spatial extent of 3D regions or it is necessary to generate mutliple 3D views. We have attempted to show images of the anatomical regions previously (2, 3). We do not feel that this informaton is necessary to present our current results and that the time and effort it would take to generate the images is not justified.

*The authors explain what D-values are in SAPM, but an explanation for B-values is missing.*

We have corrected the sentence spaning lines 305-306 to explain the B values and it now reads:

“The B values reflect how the incoming signal is converted to contribute to the output signal within each region. ”

*Which software (and its version) was used to perform statistics after SAPM, e.g. for correlations and ANCOVAs?*

The ANCOVA and correlation methods are based on mathematical formulas that do not differ between software or its versions.All statistical analyses were done in python. Correlations were computed using the “corrcoef” function in the “numpy” (version 1.23.5) module, and ANCOVAs were computed using the “anova_lm” function in the “statsmodels” (version 0.14.0) module. We do not feel that these details are useful information for the reader. Nonetheless, we have clarified how the statistical values were computed by adding the following sentence in the Methods:

“All statistical analyses were done in python using custom-written software using freely shared python modules including numpy and statsmodels.”

*For ANCOVAs the models and the results for all tested factors and interactions should be reported at least in a supplement. Additionally the models should be presented in methods. This information is necessary to assess the rigor of the statistical analyses.*

We have added the following to provide more information about the ANCOVA model that was used:

“Analyses of covariance (two-way ANCOVAs) were used to investigate how connectivity strengths (specifically DB values) varied in relation to the study groups (FM vs HC) and questionnaire scores from each participant”

We tested 52 different network connections for each questionnaire score. Reporting the results for all combinations of all tested connections and all questionnaire scores is not practical. We feel that reporting the signficant results provides a sufficient level of detail to judge the rigor of the analysis, and to effectively communicate the key results to the reader.

*In general, no “omissions for brevity” should be made without being presented in a supplement.*

There are only two places in the manuscript where we state that we show selected information “for brevity”. One is when reporting the significant connections identified with ANCOVA analyses (Table 3) and the other is when we report which factors appeared to be related to the magnitude of the initial rise on BOLD signal. In both of these instances we are comparing fMRI results (connectivity values, BOLD changes) to the behavioral data obtained from each person in the form of pain ratings and questionnaire scores. We do not see the value of reporting the results which show no relationships when they would obscure the results that appear to show significant relationships. This addition would only add significant length to the paper and supplementary material without adding to its clarity. Reducing the readability would only reduce the impact of this work.

*The authors should add a discussion of the limitations of their study.*

We have added a limitations section to the Discussion as follows:

“**Limitations**

The SAPM method is inherently limited because it models complex systems of interconnected regions of the central nervous system as a simplified linear model in order to aid our understanding of neural signaling within such systems. The linear approach is necessary in the absence of evidence for how to better model more complex non-linear systems, and represents a first-order approximation of signaling across the network. The advantage of the linear modeling approach is that it requires fewer fit parameters and is less likely to suffer from over-fitting. Moreover, with the SAPM method we model the average effects of signaling between regions/sub-regions, again in order to simplify the model, avoid over-ftting, and facilitate our understanding of the results. Another form of simplifcation is that the model is not complete, as it involves only selected regions within the volume spanned by the data, and it does not include signaling within each region. Nonetheless, the method has been shown to provide valuable insights into neural signaling across networks and highly consistent results. The results have been validated previously in relation to known properties of neural signaling. The present study shows that the SAPM approach provides a much more detailed picture of neural signaling and how it is altered in FM than can be achieved by other methods, in spite of the limitations.”

*A brief discussion whether the initial rise could be a technical artifact or not might back up the conclusions.*

We have add the following to the Discussion to address this point:

“A third possible explanation for the initial rise that must be considered is that it is the result of errors or artifacts, and is not related to physiological effects. The fact that the initial rise does not occur in two sets of results from HC participants (from the FM and PVD studies) and does not occur in the PVD group provides evidence that the initial rise is not a systematic error or artifact arising from the data acquisition or analysis methods. MRI signal variations related to changes in relaxation-time weighting (T1-weighting) at the beginning of each run produce decreases in signal over time, not increases, and were avoided by masking the data from the first three volumes of each run. Moreover, differences in this effect between the FM and HC groups would require consistent differences in the tissue properties in the brainstem and spinal cord and correspondingly different T1 values. This would be an important finding, but there is no evidence of this difference in tissue properties occuring. Finally, effects of motion artifacts, specifically in the FM group, can be ruled out as a cause because records of motion obtained from the co-registration step during the preprocessing of each fMRI run do not show any evidence of consistent motion effects in either the FM or HC groups. We therefore conclude that the observed initial rise is physiological in nature and is a BOLD signal response.”

***Reviewer #2:*** *In the manuscript “Evidence of a persistent altered neural state in people with fibromyalgia syndrome…” the authors reanalyze several prior sets of data using a novel analytic technique (SAPM). The main finding is that the main change in responsiveness noted appears related to the fMRI environment in FM more than the warning about noxious stimulation or the stimulation itself. The authors postulate, with correlation data with anxiety questionnaire measures, that this consistent with a sympathetic autonomic response to anxiety or stress throughout the fMRI acquisitions.*

*The authors suggest that the data presented in Figure 2 justify combining disparate methods together for this analysis. To my eyes, I am not sure that assumption is valid. Using some sort of metric to demonstrate intercompatibility would be desirable here.*

In order to provide more information and clarify this point we have added the following text to the description, and we have moved this information into the supplementary material:

“For example, data sets obtained with the two methods demonstrate an initial rise in signal, as well as signal variations when participants were informed of what to expect and while anticipating the stimulus. This was followed by relatively low signal variations during the stimulation period. Direct comparison of the time-series responses were obtained by first interpolating the values to the sampling rate of the brainstem/cord data (TR = 6.75 sec, 40 time points) and allowing time shifts up to ½ of a TR period to account for differences in slice timing. The time-series responses with the two methods were observed have correlation, R, values of 0.263 and 0.446 for HC and FM data, respectively (corresponding to p = 0.051 and p = 0.0018). The data therefore demonstrate similar BOLD response features and the two methods are expected to provide similar results with SAPM.”

We have also added the following sentence to the caption for supplementary Figure S1 (formerly Figure 2):

“With data interpolated to the same TR interval as for the brainstem/cord data and allowing time shifts of less than TR/2 to account for diffferences in slice timing corrections, the correlation, R, between the time-series responses with the two methods are 0.263 and 0.446 for HC and FM data, respectively (corresponding to p = 0.051 and p = 0.0018).”

*The connectivity analysis, confined to network figures and tables, seems very fair. Statistics seem appropriate and the data seems useful. The rest of the manuscript is heavy with noisy waveform figures, the purpose of which is not always easy to intuit. It is also not an easy read.*

We understand that this paper is heavy with data, but we believe that these data are necessary evidence to support our conclusions. We have made revisions to the text, as detailed below, to try to make the paper easier to understand and to better explain the results. It is an important feature of our results that they demonstrate increases, decreases, etc, at specific points in time which can be explained based on the latent inputs and the network structure across many repeated runs, and therefore cannot be considered to be “noise”. We hope that the edits we have made to the manuscript demonstrate this conclusion more clearly.

*The finding of most interest to the authors is displayed in Figure 5. The rapid increase in BOLD activity across many regions of the network discussed in Figure 1 occurs just from starting the task, independent from pain cues or pain stimulus. This is interesting and it relates to anxiety scores. It is also much greater than the changes that come with pain cues or pain stimulus, which is interesting consider recent ideas of central sensitization focus on overactive nociceptive responsiveness. The authors then observe that this pre-task BOLD increase is not a feature of a focal pain disorder (PVD). The authors then report pupil dilation data in a single line. Again, the readers are asked to eyeball the group differences without any metrics to help calculate group differences. The authors discuss that they interpret this eye tracking data to be persistent autonomic tone (perhaps constantly driving the oculomotor nerve to stay dilated); however persistent autonomic tone is not what they observed in the brain. This divergence seems to merit some further elaboration.*

***Re: Figures 4 and 5***

The original Figure 4 (now Figure 3) demonstrates that the timecourses have consistent increases in signal at the start of each run, regardless of whether or not a stimulus is applied, and that this occurs in all of the regions that were studied. The initial rise is clearly visible and is larger in the FM group. This figure also demonstrates that in runs with stimulation there are BOLD increases after the onsets and offsets of stimulation in both FM and HC groups.

The original Figure 5 (now Figure 4) then provides a more detailed comparison/contrast between the FM and HC results by showing them on the same plots, and fewer regions are shown so that the plots can be larger and more detail is visible.

Following the reviewer’s suggestions, we have revised the figures to provide more quantitive information and to attempt to better demonstrate the features within the data. We have added values to show the changes in BOLD signal intensity from the start of the run to the sustained level throughout most of the run (not counting the stimulation period, or the initial 1 minute period). The original figures showed the group averages of the SAPM fit BOLD responses in order to illustrate the consistent differences across region and study groups. In the original plots it appears that that two data sets did not have identical vertical scaling, and the separation between the two time courses is now even more evident. We have confirmed that the vertical scaling is now exactly the same for all of the plots. The revised figures show the measured BOLD responses to correspond with the values that were used for the subsequent ANCOVA analyses.

The key features of the BOLD responses that are being compared are that the initial rise is consistently larger in the FM group, and that both groups show consistent responses to being informed of the stimulus and to the application of the stimulus. In addition, these plots show that the BOLD responses are not identical in all of the regions, including different magnitudes of the initial rise. We believe that the revisions to the plots better illustrate this point.

The text has been revised accordingly to describe the data that are shown in Figures 3 and 4 (formerly Figures 4 and 5).

***Re: PVD vs HC data***

We have also added text to elaborate on the comparison of HC and PVD groups, as follows:

“The time-course responses are plotted with the same vertical scales and are shown to be highly consistent across the PVD and HC groups, with notable differences in the responses across regions. BOLD signal changes are apparent at the time that participants were informed of impending stimulus, and brief increases in signal are visible in most regions after the onset and offset of stimulation. The results in Figure 6 for the HC group are also consistent with those shown in Figure 3.”

***Re: Eye-tracking data***

We have also revised Figure 7 to add quantitive values for the average pupil areas, and reference lines as visual guides. We have revised the text by adding the following sentence:

“The average pupil area is 1540 pixel units in the HC group, and 1920 pixel units in the FM group, demonstrating that, on average, the FM group had approximately 25% larger pupil area than the HC group.”

*The authors discuss that they interpret this eye tracking data to be persistent autonomic tone (perhaps constantly driving the oculomotor nerve to stay dilated); however persistent autonomic tone is not what they observed in the brain. This divergence seems to merit some further elaboration.*

We interpret the pupil size measurements as potentially demonstrating persistent autonomic tone because it is the sympathetic nervous system that drives pupil dilation. We do not believe that it can be concluded from the BOLD responses in the brain alone whether or not they are related to persistent autonomic tone. All of the proceses involved are linked – emotional, cognitive, and autonomic – and these are all essential elements of pain processing.

We have elaborated on this in the Discussion as follows:

“The BOLD signal variations in the brain were observed to occur in a number of regions that are known to be involved with pain processing, but many of the regions shown in Figure 3 including the IC, hypothalamus, and PAG, are also components of an autonomic signaling network as described by Craig (4). The effects of altered autonomic signaling can therefore be expected to influence signaling across the entire pain network. The interconnected effects of autonomic signaling and emotional and cognitive influences cannot be distinguished in the results of the present study in terms of how they contributed to, or were affected by, the pain that resulted from the noxious heat stimuli. The observed BOLD responses and pupil measurements are both consistent with the conclusion that the results reflect heightened signaling across interconnected networks of regions in FM, compared to the HC group, during each fMRI run.”

*The authors note that this effect returns to a baseline level between fMRI runs. They do not clarify what that means in regards to the participant. Assuming that these participants remained in the magnet between runs, what was the stimulus that noted to the participant that a new run was starting. Was it a verbal cue? Was it the sound of the magnet starting up? If there is no discernable cue between runs, the finding takes on a different meaning. This warrants elaboration.*

We have added more detail to the Methods to clarify that participants provided pain ratings between each run, as practiced during the training session. Participants remained in position in the MRI system between runs and it is very apparent to the participants when a run is on-going, and when it is a time between runs. The MRI system creates loud sounds while image data are being acquired and participants were given ear plugs to diminish this noise. The visual display also provided information during the fMRI runs. The participants therefore had visual and auditory cues of when a run was starting or on-going, and they were only asked to verbally report pain ratings after each run was completed.

We have added the following text to the Methods section to be more clear:

“Participants viewed a rear-projection display while positioned inside the MRI system and were given cues as to when a run would start, information during the run about the stimulus to expect, and other than when information was being provided the displayed showed the pain rating scale.”

“Participants were reminded at the start of the fMRI session to mentally rate their pain to each contact as practiced during the training session. At the end of each run participants were asked to verbally report their pain rating for the first and last of the heat contacts.”

We have also elaborated in this point in the Discussion section as follows:

“Participants were familiarized with the study procedures prior to the MRI session, and were informed via visual and auditory cues when an imaging session was about to begin, and also by the sounds of the MRI system when imaging was in progress. While it is possible that the sounds of the MRI system during imaging evoked fear or stress and explain the rise in BOLD signal in the FM group, we note that the pain ratings to the calibrated stimulation temperatures were consistent between the training session (without any MRI sounds) and the fMRI session. Nonetheless, it is possible that each fMRI run created a mentally stressful environment for the participants.”

*The manuscript feels like two separate manuscripts at times. The BOLD results are not placed in the context of the network connections that the authors’ painstakingly drew. Does knowing the network pathways elicited earlier in the manuscript predict where the BOLD phenomenon occurs as described later in the manuscript? Conversely, what does the network of BOLD activation the authors observed look like? How do these two different types of data inform each other?*

In order to add this context to the manuscript, we have added the following sentence to the Discussion section.

“While the connectivity values demonstrate the structure of the brainstem/cord network and the influences of signaling between regions, it is the BOLD signal variations that demonstrate how this signaling varies over time and in response to external influences such as heat stimuli and emotional or cognitive influences. ”

The following has also been added near the end of the Discussion section:

“Together, the analyses of BOLD timecourse responses and connectivity values provide a detailed description of neural signaling related to pain, and how it is altered in FM. For example, the BOLD responses in the IC may be the result of latent input, possibly via afferent autonomic input as described by Craig (ref), and by excitatory input from the AC as indicated by the SAPM connectivity results. The AC🡪IC connectivity values are lower in FM than in HC participants meaning that input signaling from the ACC causes greater output signaling from the IC in the HC participants. The initial rise in BOLD signal is larger in the IC in people with higher state anxiety scores (STAI-Y-1), and the effect is more exaggerated in people with FM. Similarly, the SAPM results show evidence of the hypothalamus receiving excitatory input from the thalamus, and its effect on the hypothalamus output is reduced in FM compared to HC, and in the HC participants during the noxious stimulation condition. The hypothalamus in turn provides input signaling to the PAG and NTS and may therefore influence descending pain regulation. The initial rise in BOLD signal is also larger in the hypothalamus in people with higher state anxiety scores, regardless of whether they have FM or not.”

*One of the issues with re-analysis of data is that it is often hard for the audience to understand how the presented findings are different from the work that came before. Without requiring the audience to review each citation, it is not clear what the results of prior analysis of this data revealed. This is worth a paragraph in the discussion.*

***Re: previous results compared to current results***

In the Introduction section the 2^nd^ paragraph summarizes the key findings from the previous studies. However, to be more clear how the previous results relate to the present study we have added the following sentences:

“The data from this previous study are included in the present analysis, with different analysis methods. A parallel study in the same participants investigated the brainstem and spinal cord and observed connectivity values that were correlated with pain ratings (Ioachim 2022). Several connections also had significant differences between the FM and control groups, including the locus coeruleus (LC), hypothalamus, periaqueductal gray region (PAG), and parabrachial nuclei (PBN). These regions are known to be associated with autonomic homeostatic regulation, including fight or flight responses (5). ”

We have added the following to the first paragraph of the Discussion section:

“The results are consistent with our previous studies which indicated that there are altered motivational and affective responses in FM compared to HC (via cortical regions) and altered brain stem modulation of responses to nociceptive stimuli (via the NRM🡪C6RD pathway (1, 6, 7).”

*The authors conclude ‘The findings may relate to the well-known global hypersensitivity of FM participants’. The authors may consider going further with what their data suggests about both fibromyalgia and central sensitization. How do these data fit in with the popular idea of central sensitization that is implicated in “osteoarthritis, rheumatoid arthritis, chronic fatigue syndrome, Ehlers-Danlos syndrome, tendonopathies, headaches, spinal pain…” (Nijs, Lancet Rheumatology 2021)? Perhaps not very well.*

The findings of this study are novel and shed new light on the changes in neural signaling in FM, but we agree with the reviewer that it is not yet clear what they mean. The results suggest a strong emotional and/or cognitive component to the altered responses in FM. The evidence suggests that it is related to anxiety scores, and autonomic responses. Others have suggested that the observed apparent central sensitization may actually be caused by altered descending pain regulation.

To provide more information related to these points we have added the following to the “Stimulation Paradigm” section of the Methods”

“Repeated heat contacts were used as the stimulus in order to cause temporal summation of second pain (TSSP) and demonstrate the effects of central sensitization that are believed to occur in FM (8, 9).”

We have also added the demongraphics information as suggested by Reviewer 1 which shows that the FM participants experience the same pain as the HC group with lower stimulation temperatures.

“As described previously (1), the calibrated temperature was significantly different (p = 2.55 x 10^-5^) between the two groups and averaged 50.89 ± 1.04 °C for the HC group and 47.51 ± 2.72 °C for the FM group. The average pain ratings to the last contact in each set of 10 contacts were 38.93 ± 12.07 and 44.22 ± 12.04 on a 0-100 scale, for the HC and FM groups respectively, and were not signficantly different. The temperatures and pain ratings demonstrate the higher heat pain sensitivity in the FM group.”

These previous results support the conclusion that central sensitization is involved with FM. Our previous studies have also indicated that descending modulation process function in FM, possibly normally, but that they appear to be reacting to altered pain and are therefore different than in HC in terms of fMRI results. We have therefore also added the following to the Discussion section to address the Reviewer’s point:

“In FM participants the NRM🡪C6RD connection was consistently negative (Table 1), indicating that it provides inhibitory signaling to the cord dorsal horn, but this effect is not observed in HC. This altered descending regulation may be a response to central sensitization in the spinal cord as has been suggested previously (8, 10), and as evidenced by the higher heat pain sensitivity in FM.”

*The introduction does set the stage for this, noting all the studies in which the brain’s pain responsiveness is not particularly different between FM and HVs. These results suggest that fibromyalgia is more akin to an anxiety disorder than a pain disorder. The largest functional brain changes that occur during a pain task are unrelated to actually having pain. Persons with a more painful phenotype had less brain activation to painful stimuli compared to those with a less painful phenotype. These data do not support the idea of fibromyalgia as a disorder of hyperactivity to painful stimulation or sensitization of the pain network to incoming stimuli. It seems that being more direct about the implications of the findings would be worth considering in the discussion.*

We have added the following to the end of the Discussion section to provide more context and expand on what we believe are the implications of our results:

“The results may illustrate the fact that pain is an emotional and cognitive response to noxious stimuli and that the responses in cortical regions involve predominantly the higher-level processes of assessing the pain and its future implications (11). This assessment depends on past experiences, the person’s current situation and environment, autonomic feed-back from the body, and the intensity and modality of the noxious stimulus. Descending pain regulation is modified by these factors via the brainstem and by reciprocal feed-back between the spinal cord and brainstem. It therefore appears that in people with FM, interactions between anxiety, autonomic responses, and pain, produce a self-sustaining state of heightened sensitivity. This state may be demonstated by hypervigilence in people with FM.“

**References**

1.	Warren HJM, Ioachim G, Powers JM, Staud R, Pukall C, Stroman PW. Using Structural Equation Modeling to Investigate the Neural Basis of Altered Pain Processing in Fibromyalgia with Functional Magnetic Resonance Imaging. Medical Research Archives. 2024;12(3).

2.	Stroman PW, Ioachim G, Powers JM, Staud R, Pukall C. Pain processing in the human brainstem and spinal cord before, during, and after the application of noxious heat stimuli. Pain. 2018;159(10):2012-20.

3.	Stroman PW, Warren HJM, Ioachim G, Powers JM, McNeil K. A comparison of the effectiveness of functional MRI analysis methods for pain research: The new normal. PLoS One. 2020;15(12):e0243723.

4.	Craig AD. Interoception: the sense of the physiological condition of the body. Current opinion in neurobiology. 2003;13(4):500-5.

5.	Ioachim G, Warren HJM, Powers JM, Staud R, Pukall CF, Stroman PW. Altered Pain in the Brainstem and Spinal Cord of Fibromyalgia Patients During the Anticipation and Experience of Experimental Pain. Front Neurol. 2022;13:862976.

6.	Staud R, Godfrey MM, Stroman PW. Fibromyalgia is associated with hypersensitivity but not with abnormal pain modulation: evidence from QST trials and spinal fMRI. Front Pain Res (Lausanne). 2023;4:1284103.

7.	Ioachim G, Warren HJM, Powers JM, Staud R, Pukall C, Stroman PW. Altered pain responses in fibromyalgia during the anticipation and experience of acute pain investigated by means of functional magnetic resonance imaging in the brainstem and spinal cord. Frontiers in Neurology. 2022;Submitted.

8.	Staud R, Price DD, Robinson ME, Mauderli AP, Vierck CJ. Maintenance of windup of second pain requires less frequent stimulation in fibromyalgia patients compared to normal controls. Pain. 2004;110(3):689-96.

9.	Bosma RL, Mojarad EA, Leung L, Pukall C, Staud R, Stroman PW. FMRI of spinal and supra-spinal correlates of temporal pain summation in fibromyalgia patients. Hum Brain Mapp. 2016;37(4):1349-60.

10.	Nijs J, George SZ, Clauw DJ, Fernandez-de-Las-Penas C, Kosek E, Ickmans K, et al. Central sensitisation in chronic pain conditions: latest discoveries and their potential for precision medicine. Lancet Rheumatol. 2021;3(5):e383-e92.

11.	Price DD, Harkins SW. The affective-motivational dimension of pain. A two-stage model. APS Journal. 1992;1(4):229-39.
