# Supplementary material for: Evidence of a persistent altered neural state in people with fibromyalgia syndrome during functional MRI studies and its relationship with pain and anxiety
Source: PLoS One. 2025 Jan 24;20(1):e0316672. doi: 10.1371/journal.pone.0316672 (PMC11759356; doi:10.1371/journal.pone.0316672)
Supplement: Attachment — Submitted filename: Responses_to_Reviews from PLOS One Nov2024.docx [file pone.0316672.s005.docx]

**Responses to Reviews from PLOS One (PONE-D-24-17827R2)**

We have addressed all of the additional comments from the reviewers.

Please find the detailed responses to each point below, following the reviewers’ comments (in italics) which we have included for clarity.

**Reviewer #1:**

*The Authors have addressed most of my comments appropriately. However, I still disagree on a few points:*

*While the math behind correlations and ANCOVAs does not change, standard parameters (e.g. types of sums of squares, inclusion of an intercept) do in fact differ between software and sometimes versions. For the sake of reproducibility, functions and package versions should be provided (as is stated in the journal and linked guidelines).*

We have now added the following sentence to provide this information:

“Correlations were computed using the “corrcoef” function in the “numpy” (version 1.23.5) module, and ANCOVAs were computed using the “anova_lm” function in the “statsmodels” (version 0.14.0) module.”

*I disagree on the notion that “ Reporting the results for all combinations of all tested connections and all questionnaire scores is not practical.” or that there would be no value in reporting the results not showing any relationships. If the analyses are worth doing, the results are worth to be reported. I agree that they should not be reported in the main text, hence the suggestion to put them into a supplement.*

We have added supplementary tables (now S2 Appendix) showing detailed statistical values for all of the ANCOVA comparisons that were done.

*Minor:*

*Please provide the number of patients and the number of controls scanned in the trio configuration.*

We have now added this information as follows:

“Of the 20 FM participants included in this study, 15 were studied before the MRI upgrade, and 5 were studied after the upgrade. Of the 17 HC participants, 12 were studied before the upgrade, and 5 after.”

*Please provide the number of participants for each group in the abstract. N=x in a bracket would be sufficient.*

This information has been added to the abstract.

*Did any of the patients take opioids or benzodiazepines? If so, please add the numbers to the participants section.*

We already mention in the “Participants” section of the Methods that the participant characteristics, including medications, were discussed in detail in the original paper reporting this data set. We have now added additional information about medications, as follows:

“”Two of the 20 FM participants were taking opioid anatagonists, one was taking opioid agonists, two were taking anxiolytics, and 17 of the 20 were taking antidepressants.”

**Reviewer #3:**

*The authors responded to almost all the requests of the previous Reviewers. However, in my opinion, a clear reference to a possible practical application for patients with fibromyalgia syndrome is still missing in the conclusions of the updated manuscript. In light of the data presented, do the authors think that these results could be helpful for the future treatment of these patients? In which way? Drugs? Neurostimulation? New emerging techniques? Others?*

We do not believe that the current results provide enough information for us to comment on possible treatments or interventions. Nonetheless, the features of FM that we have identified are an important advance in our understanding of FM. We have attempted to address the reviewer’s question by adding the following sentence to the Conclusions:

“Future studies are required to determine whether or not the findings of this study are specific to people with FM, or are a common feature of chronic pain conditions, and how these findings may be used to develop improved diagnostic methods and treatments for FM.”

*I would also include among the limitations the analysis methodology used since no papers in the english literature (if we do not consider those of the authors themselves) have used this SAPM method for the analysis of fMRI datasets.*

We have added the following sentence to the limitations section:

“However, as with any new method, SAPM is still limited because it is not yet used by multiple researchers and requires further validation and testing, which is one of the purposes of the present study.”
